# Splitting random forest (SRF) for determining compact sets of genes that distinguish between cancer subtypes

**DOI:** 10.1186/2043-9113-2-13

**Published:** 2012-05-22

**Authors:** Xiaowei Guan, Mark R Chance, Jill S Barnholtz-Sloan

**Affiliations:** 1 Case Comprehensive Cancer Center, 11100 Euclid Avenue, Cleveland, OH, USA; 2 Center for Proteomics and Bioinformatics, 10900 Euclid Ave., BRB 930, Cleveland, OH, 44106-4988, USA

**Keywords:** Tree based models, High dimensional data, Cancer subtypes

## Abstract

**Background:**

The identification of very small subsets of predictive variables is an important toπc that has not often been considered in the literature. In order to discover highly predictive yet compact gene set classifiers from whole genome expression data, a non-parametric, iterative algorithm, Splitting Random Forest (SRF), was developed to robustly identify genes that distinguish between molecular subtypes. The goal is to improve the prediction accuracy while considering sparsity.

**Results:**

The optimal SRF 50 run (SRF50) gene classifiers for glioblastoma (GB), breast (BC) and ovarian cancer (OC) subtypes had overall prediction rates comparable to those from published datasets upon validation (80.1%-91.7%). The SRF50 sets outperformed other methods by identifying compact gene sets needed for distinguishing between tested cancer subtypes (10–200 fold fewer genes than ANOVA or published gene sets). The SRF50 sets achieved superior and robust overall and subtype prediction accuracies when compared with single random forest (RF) and the Top 50 ANOVA results (80.1% vs 77.8% for GB; 84.0% vs 74.1% for BC; 89.8% vs 88.9% for OC in SRF50 vs single RF comparison; 80.1% vs 77.2% for GB; 84.0% vs 82.7% for BC; 89.8% vs 87.0% for OC in SRF50 vs Top 50 ANOVA comparison). There was significant overlap between SRF50 and published gene sets, showing that SRF identifies the relevant sub-sets of important gene lists. Through Ingenuity Pathway Analysis (IPA), the overlap in “hub” genes between the SRF50 and published genes sets were *RB1, πK3R1, PDGFBB* and *ERK1/2* for GB; *ESR1, MYC, NFkB* and *ERK1/2* for BC; and *Akt, FN1, NFkB, PDGFBB* and *ERK1/2* for OC.

**Conclusions:**

The SRF approach is an effective driver of biomarker discovery research that reduces the number of genes needed for robust classification, dissects complex, high dimensional “omic” data and provides novel insights into the cellular mechanisms that define cancer subtypes.

## Background

An important challenge confronting researchers lies in analyzing large scale high-throughput “omic” datasets and interpreting results in a biologically meaningful way. Although identifying gene sets that distinguish tumor compared to normal tissue has become routine, it is more challenging to provide compact gene sets that define histological subtypes of cancers or predict outcome and/or treatment response. In addition, because sample sizes (n) are generally very small as compared to the dimensionality of the “omic” data being analyzed (i.e. “big p, small n phenomenom”), overfitting of the data and false positive results have to be carefully considered. Powerful statistical methods are in great need to rigorously validate sub sets of accurate predictors across independently collected blinded samples from different populations or data sets before these predictors could be used in clinical care [1].

As the state-of-the-art data mining techniques can fall short of being able to extract the compact gene sets from high dimensional data, feature selection techniques have been developed to prioritize a compact subset of the original features according to a specific criterion without performance deterioration. Feature selection techniques are classified into three categories: filter, wrapper and embedded, depending on how and when the utility of selected features is assessed [2]. Filter methods select features as a pre-processing step and involve no learning. These methods are not efficient because they treat biomarkers independently and ignore the interactions among them. Wrapper methods need a predetermined learning algorithm as a black box to score the selected feature subsets; while these methods account for gene interactions, they face the problems of over fitting and high computational complexity. Embedded methods build the search for an optimal subset of features in the combined space of feature subsets and hypotheses and show superior performance while taking into consideration the interactions of the genes and being less computationally intensive[3]. Linear classifiers (which fall in the embedded class), such as the least absolute shrinkage and selection operator (LASSO)[4], simultaneously produce an accurate and sparse model because of the regularization resulting from its L_1_ penalty (L_1_ regularization penalizes the weight vector for its L_1_-norm, i.e. the sum of the absolute values of the weights. However, the LASSO has several limitations one of which is that the number of selected variables is limited by the number of observations (n), a considerable limitation to finding compact gene sets [5].

Considering the above advantages and disadvantages of current analysis methods, new robust analysis methods are needed to take into account the inherent variability of individual tumors and to permit identification of genes that dominate differences between known tumor subtypes. Molecular stratification of tumors and/or individuals that reveals the activation state of specific biological pathways could allow for improvements in the diagnosis and treatment of cancer. The ensemble-based feature selection method, Random Forest (RF) [6], allows for relatively few hyperparameters, ease of identification of variable interactions- as many phenotype groups as necessary- and is robust to missing values and outliers. Hence, an extended algorithm, Splitting Random Forest (SRF), which extends the baseline single RF model, was developed. The SRF algorithm embeds a random splitting test-train technique into the standard RF algorithm, allowing for identification of a small set of genes that distinguish between groups while preserving robust classification power to accurately distinguish between tumor subtypes. SRF offers a minimal requirement of human input, robust predictive performance, and low computational cost.

## Methods

### Algorithm

The SRF approach is an interactive, non-parametric, decision tree-based classification statistical method built on an ensemble of standard un-pruned classification and regression trees (CART) utilizing all information on all genes[7][8] (i.e. a “forest” or the Random Forest (RF) method) in order to identify the optimal tree. The individual decision trees are generated *via* bootstrap samples of the original data which is used as the “training” set from which to grow the tree. Part of the original data is not sampled and is used as a “testing” set for the tree; this group of data is called the “out of the bag” (OOB) samples. The OOB samples‘error rate is calculated based on the number of trees in the forest and this rate is generated for each gene to gene sub-cluster. The smaller the OOB error rate, the better the classifier. Class prediction is made by polarity voting for classification for one of the subtype grouπngs and variables for this classification are selected based on their variable importance values (VIM).

Let ${\bar{\beta }}^{\left(t\right)}$be the out-of-bag sample for a tree $t(i=\text{1 … }n_{\mathrm{tree}})$, RF employs the following steps to calculate the importance value $z_{x_{j}}$for the variable $x_{j}$ in the tree *t*[9]:

(a) Classify the OOB samples from the tree and record the number of the correctly predicted classes, denoted as:

(1) $$N_{original,x_{j}}=\frac{\sum_{i\in {\bar{\beta }}^{\left(t\right)}}I(y_{i}=y^{'}{{}_{i}}^{\left(t\right)})}{\left|{\bar{\beta }}^{\left(t\right)}\right|}$$

where $y^{'}{{}_{i}}^{\left(t\right)}=f^{\left(t\right)}(X_{i})$is the predicted classes for observation *i* before permuting the values of variable$x_{j}$across all samples, where $X_{i}=(x_{i,1},\ldots ,x_{i,j-1},x_{i,j},x_{i,j+1},\ldots ,x_{i,p})$.

(b) Randomly permute the values of the predictor variable $x_{j}$ across all samples with respect to *Y*. Rerun the analysis described in step (a), using the permuted variable,$x_{j}$, together with the remaining non-permuted predictor variables, to predict the response for the OOB observations. The recorded number is denoted as:

(2) Npermuted,xj= ∑ i ∈  β ¯(t)I(yi=y'i, π j(t)) β ¯(t)

where y'i, π j(t)=f(t)(Xi, π j)is the predicted classes for observation *i* after randomly permuting the values of variable $x_{j}$, where Xi, π j=(xi,1, … ,xi,j−1,x π j(i),j,xi,j+1, … ,xi,p). For the vector of *p* values associated with the observation*i*, only the value for the $x_{j}$ variable is the randomly permutated value and all other p−1 values for the p−1 variables remain unchanged.

(c) Calculate the difference of the two counts, denoted as:

(3) $$N_{original,x_{j}}-N_{permuted,x_{j}}$$

It will decrease substantially if the original variable $x_{j}$ is associated with the response *Y*. By definition, this value is 0 if variable $x_{j}$ is not in tree *t*.

(d) Calculate the non-scaled variable importance measurement (VIM), average over all trees, defined as:

(4) $$z_{x_{j}}=\frac{1}{n_{\mathrm{tree}}}\sum_{i=1}^{n_{\mathrm{tree}}}\left(N_{original,x_{j}}-N_{permuted,x_{j}}\right)$$

This algorithm employs the non scaled VIM, which means the VIM value isn’t divided by the standard error (SE) of the VIM, denoted as:

(5) scaledVIM=VIMSE(VIM)

The non-scaled variable importance measurement (VIM) for regression was adopted here as it has been reported that scaled variable importance measurement was dependent on forest size and predictor correlation. [9-11]. After each iteration, variables with the lowest importance values are removed and a new forest is generated using the remaining variables. Individual trees provide the user with clear information about hierarchy and relationships between factors; genes at the top of the tree are the most important and each connection within the tree links various combinations of genes together.

**Figure 1:**
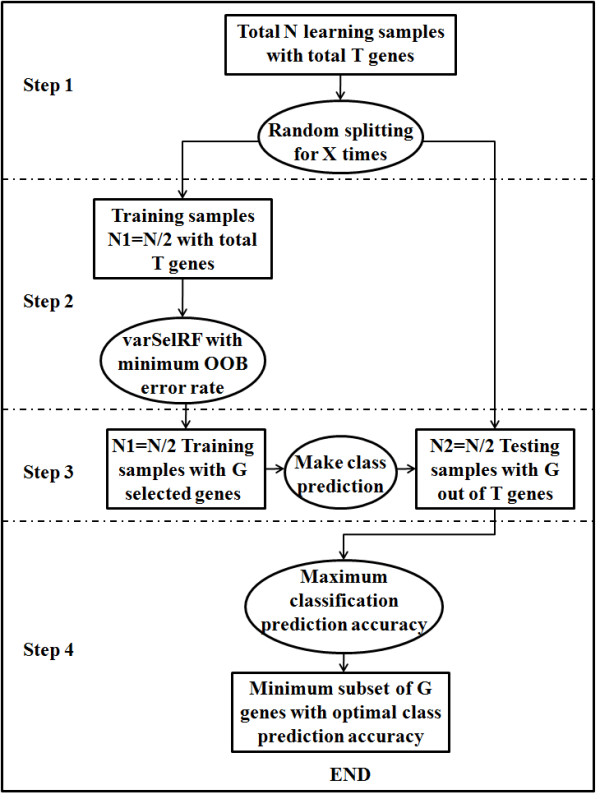
Flowchart of the Splitting Random Forest (SRF) Algorithm.

SRF builds upon the RF algorithm by providing an iterative sampling of the data to investigate a wide landscape of solutions in order to find the one that optimally distinguishes between cancer subtypes rather than having to use predefined cut off values of variable importance. SRF employs four steps as follows (Figure 1):

**Step 1**: Starting with a high-throughput dataset, the time (X) is set as the total number of times that the data use randomly split into training and testing datatests. RF is then run on the training datasets. For each run, the original samples N are split into equal halves. One half becomes the training dataset (N1 = N/2) and the other becomes the testing dataset (N2 = N/2) for each iteration.

**Step 2**: Based on the training dataset for each iteration, the RF variable selection package from R, varSelRF [12] is then used to identify the optimal subset of genes (G) out of the total genes (T) with the minimum OOB error. The number of optimal genes, G, could vary between different iterations. In total, X sets of optimal genes are generated according to varSelRF algorithm.

**Step 3**: For each iteration, the gene set (G) is then used as a classifier to make class predictions on the matching testing dataset and the overall prediction accuracy of the classifications is calculated based on the true classes of the testing data, which is the ratio between the number of true positives across all subtypes and the total sample. A set of X prediction accuracies are generated.

**Step 4**: Finally, the maximum overall prediction accuracy is selected based on the maximum value from all X prediction accuracies and the gene set associated with this maximal prediction accuracy is extracted as the optimal set that distinguishes between cancer subtypes.

X was set at 50, 100 or 500 runs in order to find the optimal run time in terms of computation cost, prediction accuracy and the number of selected predictors. By embedding the splitting in the RF algorithm, the risk of overfitting is minimized. As SRF is a modified extension of the varSelRF method, we utilize the default parameter settings for robust performance. The parameters *ntree* and *nodesize* were set at 5000 and 1, respectively, as these values have been shown to be robust. The default, *fraction. dropped* = 0.2 allows for relatively fast computational time, which is in coherent with the “aggressive variable selection” approach[11]. Since the SRF algorithm applies a test-train technique upon the varSelRF algorithm, computational time is just a linear rate of that of varSelRF according to how many splits are utilized. R and Bioconductor [13,14] were used for all data management and statistical analysis. R code for the SRF algorithm is available at http://epbiwww.case.edu/SRF.

### Data sets

Three publicly available datasets were used: glioblastoma, breast cancer and ovarian cancer (details are described below). The original normalized values, without further renormalization or filtering, were maintained for the evaluation of SRF so as to effectively compare them with the intrinsic gene lists as identified in the publications for these cancers.

### Glioblastoma (GB) datasets

Publically available data from The Cancer Genome Atlas (TCGA) project as described in Verhaak et al. [15], was used for analysis, including whole-genome gene expression data on 173 individuals. Individuals were divided into the following four known GB gene expression-based subtypes identified by a consensus clustering algorithm: proneural (N = 53), neural (N = 26), classical (N = 38), and mesenchymal (N = 56) [15]. Three datasets generated from these data were used for analysis: (1) a unified dataset with information on 11,861 genes after merging the gene expression data from Affymetrix Human Exon 1.0 ST GeneChips, Affymetrix HT-HG-U133A GeneChips and custom designed Agilent arrays as described in Verhaak et al. [15]; (2) a dataset including only the 840 genes selected using SAM [16], and ClaNC [17] methods as the intrinsic GB subtype classifier by Verhaak et al. [15] (i.e. Verhaak dataset) and (3) a validation dataset where gene expression array information on 176 individuals was integrated from three studies, Beroukhim et al. [18], Phillips et al. [19] and Sun et al. [20], using the Affymetrix HG-U133A or HG-U133plus2 GeneChip platforms.

### Breast cancer datasets

The Netherlands Cancer Institute (NKI-295) oligonucleotide microarrays breast cancer dataset was used for analysis[21]. Breast cancer subtypes were assigned using consensus clustering based on the intrinsic gene list of 979 genes found in four previous microarray studies as reported by Parker et al. [22-26]. Data on 172 samples was available, these were further separated into 91 training samples (basal-like (N = 29), HER2 (N = 10), luminal A (N = 21) and luminal B (N = 31)) and 81 validation samples (basal-like (N = 18), HER2 (N = 11), luminal A (N = 20) and luminal B (N = 32)).

### Ovarian cancer datasets

Gene expression data from Affymetrix U133 plus 2.0 array platform as reported by Tothill et al. [27] was utilized in the analysis. Optimal consensus k-means clustering using 285 annotated serous and endometrioid ovarian cancer samples identified six novel molecular subtypes (C1-C6); C1-serous with low malignant potential, C2- high grade ovarian cancer with a high immune signature, C3- low malignant potential, C4- high grade ovarian cancer with low stromal response, C5-high grade ovarian cancer, mesenchymal, low immune signature and C6-low grade endometrioid. The vast majority of high grade serous and endometrioid ovarian cancer samples (N = 215) segregated with four of the high grade clustering predictions generating an intrinsic gene classifier that included 2,107 genes identified using SAM[15] in a one-versus-rest fashion [16]. The ovarian dataset was further separated into 107 training samples: C1 (N = 41), C2 (N = 25), C4 (N = 23) and C5 (N = 18) and 108 validation samples: C1 (N = 42), C2 (N = 25), C4 (N = 23) and C5 (N = 18).

### Additional methods

Fisher’s exact tests were carried out to assess performance of SRF after 50, 100 and 500 runs based on the true positive rates of subtypes within GB, BC and OC data. Non-significant Fisher’s exact p-values (0.9984, 0.9999 and 0.4057) for GB, BC and OC, respectively) demonstrate comparability among the three SRF runs (additional file 1: Tables S 1, S 2, S 3, S 4, S 5, S 6, Additional file 1: Figures S 1, S 2, S 3, S 4). Hence, SRF after 50 runs (SRF50) was used in each dataset to determine overall prediction accuracy, subtype prediction accuracy and pairwise area under the curve (AUC) as compared with four traditional statistical methods: single run of varSelRF algorithm (“Single RF”), ANOVA, Top 50 ANOVA and published gene sets. Single RF denotes a single run of random forest variable selection method using the varSelRF R package[12]. ANOVA involves two steps, an ANOVA test to discover the dominant genes between cancer subtypes and a further false discovery rate (FDR) correction [28] to account for multiple comparisons. Top 50 ANOVA refers to the Top 50-genes extracted from the above ANOVA results after ranking the genes based on the FDR corrected p-values. The published gene sets are the “intrinsic” gene lists obtained from published classifiers of the molecular cancer subtypes for each cancer of interest. For the sake of comparison, each of the five methods was evaluated using validation datasets originally used per each cancer’s corresponding publication. Classifiers generated from each method, SRF50, Single RF, ANOVA, Top 50 ANOVA and published gene sets, was used to build random forests on the validation datasets for GB, BC and OC.

With respect to performance evaluation criteria, overall prediction accuracy is the ratio of all the true positives of each subtype to the total sample, whereas subtype prediction accuracy is the ratio of the number of true positives for each subtype to the total number of cases of that subtype. The robustness of the prediction accuracy was evaluated *via* pairwise AUC comparisons using two composite measurements: the multi-class AUC value (the average of all of the pairwise AUC values) and the Area Covered by Radar Chart (ACRC) value[29]. Finally, the SRF50 genes were further investigated for biological pathway connections and functions using Ingenuity Pathway Analysis (IPA) (http://www.ingenuity.com) and were compared with the intrinsic gene lists. “Hub” genes were defined as a gene within a top scoring network that had at least five associated genes within that network. Those hub genes were further investigated through a literature review to gain more biological insight into the cancer subtypes.

## Results

### Glioblastoma (GB) results

**Figure 2:**
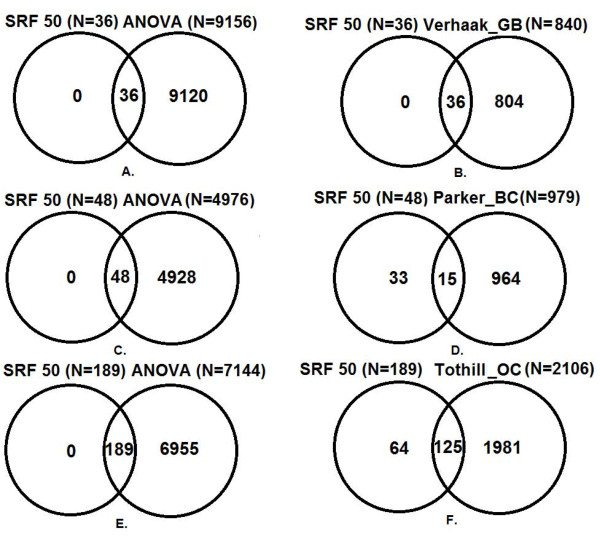
**Venn Diagrams of the overlap between the SRF50, published datasets and the ANOVA gene lists.A.** &**B.** for GB; **C.** &**D.** for breast cancer (BC) and **E.** &**F.** for ovarian cancer (OC).

SRF50 on the full GB dataset found a compact set of genes (N = 36) with high predictive accuracy (95.4%; Table 1 GB). All of the genes selected by SRF50 were included on the list of significant genes by ANOVA after a FDR correction at the significance level of 0.01 (Figure 2A) and were included as part of the Verhaak classifier (Figure 2B). However, the decrease in the sizes of the relevant gene sets is substantial, ANOVA identified 9,156 genes, Verhaak identified 840, single RF identified 88, and SRF50 identified 36.

**Table 1:** Comparison of SRF running times (50, 100 and 500) in the GB full dataset, the BC training and the OC training datasets

**Cancer**	**Running Experiment**	**Maximum Accuracy Rate (Proportion of Increase)**	**No. of Genes**
GB	50	95.4% (.)	36
	100	96.6% (+1.2%*)	23
	500	98.9% (+3.5%**)	29
BC	50	93.6% (.)	48
	100	93.6% (+0%*)	50
	500	95.7% (+2.1%**)	32
OC	50	92.7% (.)	189
	100	94.5% (+1.8%*)	290
	500	94.5% (+1.8%**)	188

*Comparison between 50 vs. 100 runs; ** Comparison between 50 vs. 500 runs.

The prediction power of the five gene lists (SRF50, single RF, Verhaak, FDR corrected ANOVA and Top 50 ANOVA) were compared for overall and subtype prediction accuracies of GB subtypes using the GB validation dataset (Table 2 GB, Table 3 GB). Of the 9,156 genes identified using ANOVA on the unified full dataset, only 8,670 genes were contained in the validation dataset. This is because data from different array platforms were merged to make this dataset (described in detail in Verhaak et al.)[15]. Similarly, 833 out of 840 genes were identified from the Verhaak gene list in the validation dataset. SRF50 achieved a prediction accuracy of 80.1% on the validation dataset. In contrast, single RF obtained a 2.3% lower prediction accuracy and used twice the number of genes as compared to SRF50. While the Verhaak gene list obtained a 5.9% higher prediction accuracy, it required 23 times as many genes as the SRF50 gene set. Likewise, ANOVA also obtained a slightly higher prediction accuracy while utilizing information on 240-times the number of genes. The Top 50 ANOVA genes generated a 2.9% lower prediction accuracy than the SRF50 set.

**Table 2:** Overall performance comparison of 5 gene lists in the GB, BC and OC validation datasets

**Cancer**	**Methods**	**No. of Genes**	**Prediction Accuracy**	**Change in Prediction Accuracy**^**+**^	**Multi-Class AUC**^**++**^	**Area Covered by Radar Chart**^**+++**^
GB	SRF50	36	80.1%		0.87	1.68
	Single RF	88	77.8%	−2.3%	0.86	1.63
	Verhaak et al.	833	86.0%	5.9%	0.92	1.91
	ANOVA	8670	84.1%	4.0%	0.9	1.83
	Top 50 ANOVA	50	77.2%	−2.9%	0.87	1.71
BC	SRF50	48	84.0%		0.91	1.85
	Single RF	46	74.1%	−9.9%	0.87	1.69
	Parker et al.	979	89.0%	5.0%	0.89	1.78
	ANOVA	4976	85.2%	1.2%	0.86	1.65
	Top 50 ANOVA	50	82.7%	−1.3%	0.87	1.72
OC	SRF50	189	89.8%		0.96	2.06
	Single RF	245	88.9%	−0.9%	0.96	2.06
	Tothill et al.	2106	91.7%	1.9%	0.97	2.11
	ANOVA	7144	90.7%	0.9%	0.97	2.09
	Top 50 ANOVA	50	87.0%	−2.8%	0.95	2.01

^**+**^All changes of prediction accuracy were calculated using SRF50 as the referent; ^**++**^ Multi-class AUC values were calculated by taking average of the all pairwise AUC values; ^**+++**^ Area covered by radar chart (ACRC) [29].

**Table 3:** Subtype prediction accuracy of 5 gene lists in the GB, BC and OC validation datasets

**Cancer**	**Methods**	**Classical (N = 50)**	**Mesenchymal (N = 48)**	**Neural (N = 30)**	**Proneural (N = 48)**
GB	SRF50	70.0%	81.3%	**73.3%**	93.8%
	Single RF	**64.0%**	77.1%	**80.0%**	91.7%
	Verhaak et al.	88.0%	91.7%	50.0%	100.0%
	ANOVA	88.0%	91.7%	40.0%	100.0%
	Top 50 ANOVA	66.0%	83.3%	73.3%	87.5%
		**Basal-Like (N = 18)**	**HER2 (N = 11)**	**Luminal A (N = 20)**	**Luminal B (N = 32)**
BC	SRF50	100.0%	**63.6%**	80.0%	84.4%
	Single RF	88.9%	**72.7%**	**55.0%**	87.5%
	Parker et al.	100.0%	54.5%	90.0%	93.8%
	ANOVA	100.0%	36.4%	85.0%	93.8%
	Top 50 ANOVA	100.0%	**63.6%**	75.0%	84.4%
		**C1 (N = 42)**	**C2 (N = 25)**	**C4 (N = 23)**	**C5 (N = 18)**
OC	SRF50	97.6%	88.0%	73.9%	**94.4%**
	Single RF	97.6%	88.0%	78.3%	**83.3%**
	Tothill et al.	97.6%	88.0%	87.0%	88.9%
	ANOVA	100.0%	88.0%	78.3%	88.9%
	Top 50 ANOVA	95.2%	88.0%	73.9%	**83.3%**

**Figure 3:**
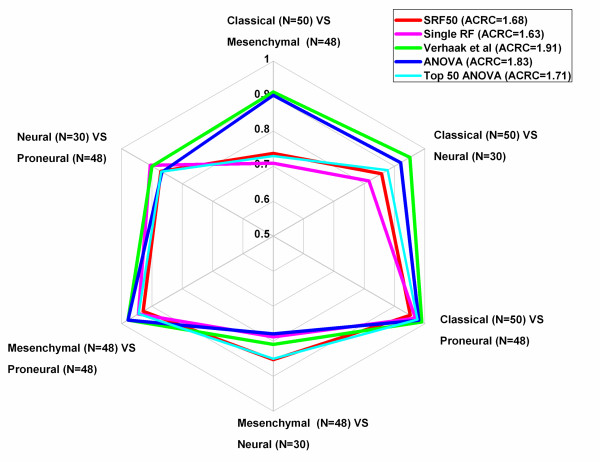
Radar Chart for pairwise comparison (AUC values) for the GB dataset.

With respect to subtype prediction accuracy, while single RF achieved the highest subtype prediction accuracy in the most undersized class (Neural, N = 30, 80.0%), it exhibited the poorest performance in the class with the highest sample size class (Classical, N = 50, 64.0%). However, SRF50 obtained a consistently robust performance across all four subtypes (70.0%-93.8%; Table 3 GB & Additional file 1: Table S 7; Additional file 1: Figure S 1). Moreover, SRF50 obtained superior results compared to single RF in both the multi-class AUC comparison (0.87 vs 0.86, Table 2 GB & Additional file 1 Table S 8GB) and the Area Covered by Radar Chart (ACRC) comparison (1.68 vs 1.63, Table 2 GB & Figure 3). Hence, SRF50 provided highly accurate results concerning both overall and subtype prediction accuracies while identifying a compact set of optimal genes for GB subtypes.

The SRF50 GB genes were further explored using IPA for biological pathway connections and functions. Additional file 1: Table S 9 (GB) lists all “hub” genes by dataset. Several of these SRF50 “hub” genes overlapped with the “hub” genes derived from the Verhaak gene list (*RB1, πK3R1, PDGFBB* and *ERK1/2*) and are known to be involved in gliomagenesis [30-33] Additional file 1: Figures S 6A & S6B).

### Breast cancer results

SRF50 on the full breast cancer dataset found a compact set of genes (N = 48) with high predictive accuracy (93.6%; Table 1 BC). All of the genes selected by SRF50 were contained on the list of significant genes found using ANOVA after an FDR correction at the significance level of 0.01 (Figure 2C). There were 15 (31.3% of the SRF gene list) common genes between the SRF50 gene list and the list of significant genes in the Parker et al. [22] dataset (Figure 2D). However, the decrease in the sizes of the relevant gene sets is considerable, ANOVA identified 4,976 genes, Parker identified 979, single RF identified 46 and SRF50 identified 48.

The prediction power of the five gene lists (SRF50, single RF, Parker, FDR corrected ANOVA and Top 50 ANOVA) was compared for both the overall and subtype prediction accuracies in the breast cancer validation dataset (Table 2 BC, Table 3 BC). SRF achieved a prediction accuracy of 84.0% on the validation dataset. As a comparison, single RF obtained a 9.9% lower prediction accuracy with a similar number of genes. Although the Parker gene list obtained a 5.0% higher prediction accuracy it required 20 times the number of genes as compared to SRF50. Similarly, ANOVA obtained a 1.2% higher prediction accuracy while requiring over 100-times the number of genes. The Top 50 ANOVA genes produced a 1.3% lower prediction accuracy than SRF50.

**Figure 4:**
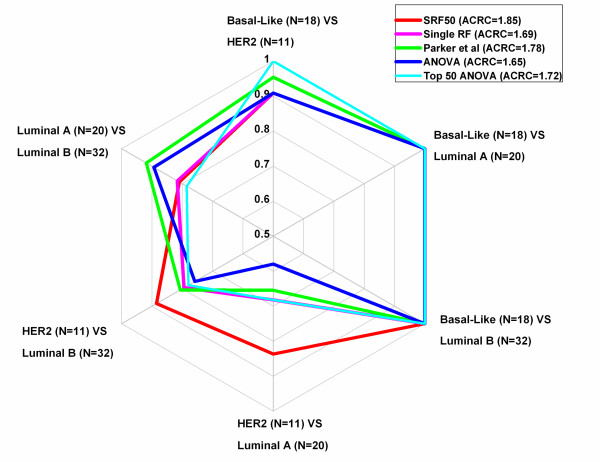
Radar Chart for pairwise comparison (AUC values) for the BC dataset.

Regarding comparison of subtype prediction accuracy, single RF achieved the highest subtype prediction accuracy in the least represented subtype HER2 (N = 11, 72.7%), yet it obtained the lowest prediction accuracy among all five methods in both Basal-Like (N = 18, 88.9% compared with 100% in all four of other methods) and Luminal A (N = 20, 55.0%) subtypes. The SRF50 gene list and the Top 50 ANOVA gene list achieved the second highest subtype prediction accuracy of the underrepresented subtype (HER2, N = 11, 63.6%) in contrast to 54.5% using the Parker et al. gene list and 36.4% from the overall ANOVA gene list(Table 3 BC, Additional file 1: Table S 10 & Additional file 1: Figure S 7). Furthermore, SRF50 achieved the best results when compared with all four of the other traditional methods in both the multi-class AUC comparison (Table 2 BC & Additional file 1: Table S 8 BC) and the ACRC comparison (Table 2 BC & Figure 4). Thus, SRF50 provided highly accurate results for both overall and subtype prediction accuracies while being robust across the subtype and identifying a compact set of optimal genes.

The SRF50 genes were further analyzed for biological pathway connections and functions using IPA. These results were compared with those using the Parker gene list. Additional file 1: Table S 9 (BC) lists all “hub” genes by dataset. In addition, a few of these SRF50 “hub” genes overlapped with the “hub” genes derived from the Parker gene list (*ESR1, MYC, NFkB* and *ERK1/2*) (Additional file 1: Figures S 8A, B & C).

### Ovarian cancer results

The optimal SRF50 gene set on the full ovarian cancer dataset found a set of probes (N = 189) with high predictive accuracy (92.7%; Table 1 OC), particularly for the high grade serous and endometrioid subtypes. All of the genes selected by SRF50 were included on the list of significant genes found using ANOVA after an FDR correction at the significance level of 0.01 (Figure 2E) and there were 125 (66.1% of the SRF50 gene list) common genes between the SRF50 gene list and the Tothill gene list [27] (Figure 2F). However, the decrease in the sizes of the relevant gene sets is extraordinary, ANOVA identified 7,144 genes, Tothill identified 2,106, single RF identified 245, and SRF50 identified 189.

**Figure 5:**
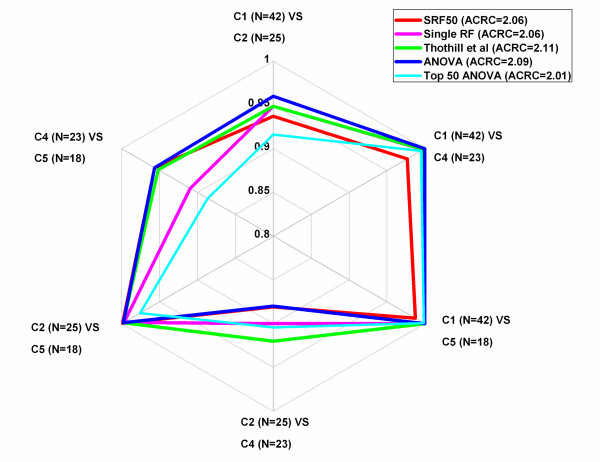
Radar Chart for pairwise comparison (AUC values) for the OC dataset.

The prediction power of the five gene lists (SRF50, single RF, Tothill, FDR corrected ANOVA and Top 50 ANOVA) were compared in terms of their overall and subtype prediction accuracies in the ovarian validation dataset (Table 2 OC and Table 3 OC). SRF achieved a prediction accuracy of 89.8% on the validation dataset. Single RF obtained a 0.9% lower prediction accuracy with 1.2 times the number of genes as compared to SRF50. Although the Tothill gene list obtained a 1.9% higher prediction accuracy it required over 11-times the number of genes as compared to SRF50. Similarly, ANOVA obtained a 1.0% higher prediction accuracy while requiring almost 40-times the number of genes. When comparing the subtype prediction accuracy using the validation dataset, the SRF50 gene list yielded the highest accuracy of the under sampled subtype (C5, N = 18, 94.4%) compared with 88.9% using the Tothill et al. gene list and 88.9% from the ANOVA gene list (Table 3 OC, Additional file 1 Table S 11 & Additional file 1: Figure S 9). Single RF and Top 50 ANOVA yielded the lowest subtype prediction accuracy, 83.3%, in the least sampled class (C5, N = 18). SRF50 achieved the same performance level through both the multi-class AUC comparison (Table 2 OC& Additional file 1: Table S 8 OC) and the ACRC comparison (Table 2 OC & Figure 5). Therefore, SRF50 provided highly accurate results while identifying a compact set of optimal genes in terms of overall and subtype prediction accuracies.

The SRF50 genes were further analyzed for biological pathway connections and functions using IPA. These results were compared with those using the Tothill gene list. Additional file 1 Table S 10 (OC) shows all “hub” genes by dataset. A number of these SRF50 “hub” genes overlapped with the “hub” genes derived from the Tothill gene list (*Akt, FN1, NFkB, PDGFBB* and *ERK1/2*) (Additional file 1 Figures S 10A, B, C & D).

## Discussion

An expanded random forest algorithm, Splitting Random Forest (SRF), was developed to discover the most compact set of genes that can distinguish between multiple groups of individuals with known cancer molecular subtypes (glioblastoma, breast and ovarian). The maximum accuracy rates of the optimal gene sets chosen from three training datasets for three types of cancer were determined using SRF with 50 runs (SRF50). The accuracy rates were similar to those derived from using the intrinsic gene lists from published molecular classifiers for each respective cancer. All three intrinsic gene lists were generated with analysis methods that are vulnerable to selection bias, prone to false positives and costly in terms of time and resources. Alternatively, SRF discovered small yet efficient sets of genes (less than 40 genes for GB and less than 50 genes for BC) that robustly distinguish between cancer subtypes and were validated in independent datasets. SRF was able to extract strikingly fewer genes in contrast to ANOVA or published gene lists for the three cancers studied. These results reveal that the SRF algorithm can identify a compact set of genes that robustly classifies cancer subtypes without requiring extensive filtering or pre-processing of the data. In addition, a multiple comparison correction is not needed when using SRF since the SRF method, by definition, prioritizes genes by importance and removes genes from the list based on the combination of a minimum build-in OOB error rate and an outer test-train prediction validation to avoid potential false positives and false negatives. The 2-fold equal split maintains robustness of the prediction accuracy and reduces the computational complexity while focusing on a compact set of genes.

The optimal SRF50 gene lists are efficient classifiers for GB, breast cancer and ovarian cancer subtypes, given that the classification prediction rates are roughly comparable with those from the published gene lists (80.1%-86.0% for GB, 84.0%- 89.0% for breast cancer and 89.8%-91.7% for ovarian cancer with those from ANOVA in the middle of the ranges) upon validation using the corresponding validation datasets from these publications. In terms of achieving optimal compact sets of classifiers with sound prediction accuracies, SRF50 achieved higher prediction accuracies than the corresponding single RF results (80.1% vs 77.8% for GB; 84.0% vs 74.1% for BC; 89.8% vs 88.9% for OC). Meanwhile, SRF50 achieved consistently higher prediction accuracies than Top 50 ANOVA genes for all three of the datasets (80.1% vs 77.2% for GB; 84.0% vs 82.7% for BC; 89.8% vs 87.0% for OC). Hence, the prediction accuracy for SRF is high regardless of sample size while utilizing information from 10–200 fold fewer genes than published classifiers. From the multi-class AUC values and the ACRC results, SRF50 outperformed or was equivalent to the single RF and the Top 50 ANOVA results in the GB and BC datasets. SRF50 achieved the same performance as single RF and outperformed the Top 50 ANOVA method in the ovarian cancer dataset. While the choice of using the Top 50 ANOVA genes may seem arbitrary and may not necessarily represent the most compact gene classifier [11]. FDR corrected ANOVA generates a long list of significant genes based on the traditionally accepted significance level of 0.05 and these longer lists are not practically useful for diagnostic purposes in clinical practice. Overall, SRF50 led to stable and robust prediction accuracies in terms of the overall and subtype prediction accuracies and exhibited a roughly similar range when compared to the published classifiers that distinguish the known molecular subtypes.

SRF provides a nested-loop validation to derive the optimal classifiers based on the maximum prediction accuracy, which generating trees *via* RF and then using varselRF would not achieve. The RF algorithm by definition has a built-in inner validation and the splitting test-train technique of SRF adds an additional outer validation to the algorithm. SRF randomly splits the data X times and then generates N tress for each split on the training dataset, and tests each result in its own training set and the optimal classifier is chosen from the results from these training sets; on the other hand RF using varSelRF would first generate X*N trees and then calculate the optimal classifier. Also, the variable importance measurement (VIM) is the average of all trees (X*N) for RF using varSelRF while for SRF the VIM is calculated based on the training dataset only which is independent of the testing dataset for any split.

The Random Forest algorithm is able to deal with large scale data when the number of variables is much larger than the number of observations (“big p small n problem”) without assuming complex models or explicitly testing all possible interactions. RF is not limited by the total number of prediction classes. RF returns measures of variable importance, which reflects the total decrease in node impurities from splitting on the specific gene, averaged over all trees. This allows for the prioritization of genes from each RF run for classification of groups, i.e. optimal gene lists. However, a single run of RF using varSelRF might identify multiple solutions based on the build-in OOB. Hence, the SRF algorithm, takes advantage of randomly splitting the original samples proportionally to the size of multiple phenotypes at multiple running times and deriving the prediction rate from one half of the data (testing dataset) based on the first half of the data (training dataset). This approach allows for an outer validation combined with the inner validation based on OOB from the varSelRF algorithm. This test-train method has become a standard statistical technique used for discovery and validation of new molecular classifiers. Future work may involve applying the random splitting test-train technique to other filter, wrapper or ensemble feature selection algorithms to potentially increase computational efficiency and accuracy as well as incorporating known biological and clinical factors into the algorithm. Other variable measurements, such as a minimum depth that assesses the productiveness of a variable by its depth relative to the root node of a tree, could also be implemented as an alternative in this setting for regularizing forests[34]. In addition, SRF could be easily extended to integrate multiple different types of high-dimensional heterogeneous “omic” data in order to gain a systems biology view of cancer subtypes. SRF could also be used as a useful means to validate sub sets of accurate predictors across independent studies [1].

In order to compare the prioritized gene lists from the SRF algorithm with those from the published gene sets in terms of gaining better biological insight, we further investigated “hub” genes with IPA. These “hub” genes may be potential key masters of the signaling or functional pathways for these cancers (Table 4 &Additional file 1 Table S 9). The common hub genes between the SRF 50 and Verhaak GB gene lists were *RB1, πK3R1, PDGFBB and ERK1/2*. *Via* IPA, *RB1* and *πK3R1* were associated with cell morphology, hematological system development and function; and PDGFBB and ERK1/2 were associated with tumor morphology, nervous system development and function, tissue morphology. The common hub genes between the SRF 50 and Parker breast cancer gene lists were *ESR1, MYC, NFkB* and *ERK1/2*. *Via* IPA, *ESR1* and *NFkB* were linked to developmental disorder, reproductive system disease, cellular growth and proliferation; *MYC* was linked to cancer, infection mechanism, gene expression and tumor morphology; and *ERK1/2* was linked to molecular transport, protein trafficking and cell cycle. The overlapπng hub genes between the SRF 50 and Tothill gene lists were *Akt, FN1, NFkB, PDGFBB* and *ERK1/2*. *Via* IPA, *Akt* was associated with antigen presentation, cell-to-cell signaling and interaction, cellular growth and proliferation; *FN1* with connective tissue disorders, genetic disorder and cellular assembly and organization; and *NFkB* with embryonic development and organismal development. *PDGFBB* with cardiac damage, organismal injury and abnormalities; and *ERK1/2* was associated with cell morphology, connective tissue development and function. The “hub” genes discovered by SRF that overlap the “hub” genes from the published intrinsic gene lists are known to be fundamental for tumorgenesis and cancer development in general. Hence, the derived SRF gene lists can help to elucidate complex cancer systems. The non-overlapπng “hub” genes could also be potential targets for improved cancer diagnosis and treatment.

**Table 4:** Biological functions identified from IPA using overlapπng hub gene lists of SRF 50 and the published sets in the GB, BC and OC datasets

**Cancer**	**Gene**	**Function**
GB	*RB1, πK3R1*	cell morphology, hematological system development and function
	*PDGFBB, ERK1/2*	tumor morphology, nervous system development and function
BC	*ESR1,NFkB*	developmental disorder, reproductive system disease, cellular growth and proliferation;
	*MYC*	cancer, infection mechanism, gene expression and tumor morphology
	*ERK1/2*	molecular transport, protein trafficking and cell cycle
OC	*Akt*	antigen presentation, cell-to-cell signaling and interaction, cellular growth and proliferation
	*FN1*	tissue disorders, genetic disorder and cellular assembly and organization
	*NFkB*	embryonic development and organismal development
	*PDGFBB*	cardiac damage, organismal injury and abnormalities
	*ERK1/2*	cell morphology, connective tissue development and function

## Conclusions

In conclusion, the SRF algorithm proves to be an effective and promising tool to identify compact sets of genes that robustly distinguish between different classes of individuals. This procedure does not require any pre-filtering and/or pre-selection procedures thus reducing the potential for bias and/or spurious findings. In addition, application of the SRF algorithm to the three types of cancer data showed that relatively small set of genes as identified by SRF had similar classification rates as compared to published classifiers. These small gene set classifiers can be investigated further as potential molecular diagnostics which could lead to tailored cancer treatments.

## Competing interests

The author(s) declare that they have no competing interests.

## Authors’ contributions

XG conceived of the study and performed all data management, data analysis, interpretation of results and drafted the manuscript. MRC assisted with interpretation of results and drafting of the manuscript. JSBS conceived of the study and assisted with interpretation of results and drafting of the manuscript. All authors read and approved the final manuscript.

## Supplementary Material

Additional file 1 **Table S1** Comparison of performance by subtype of SRF50, 100 and 500 runs in the GB full dataset. **Table S2** Comparison of performance by subtype of SRF50, 100 and 500 runs in the breast cancer training dataset. **Table S3** Comparison of performance by subtype of SRF50, 100 and 500 runs in the ovarian cancer training dataset. **Table S4** Comparison of the gene lists from SRF50, 100 and 500 runs for GB. **Table S5** Comparisons of the gene lists from SRF50, 100 and 500 runs for breast cancer. **Table S6** Comparisons of the gene lists from SRF50, 100 and 500 runs for ovarian cancer. **Table S7** Comparison of performance by subtype of five gene lists in the GB validation dataset. **Table S8** Pairwise AUC comparison between subtypes of five gene lists in the GB, BC and OC validation dataset. **Table S9** Hub genes in the top networks from IPA analysis for the SRF50 and published gene lists for GB, breast cancer (BC) and ovarian cancer (OC). **Table S10** Comparison of performance by subtype of five gene lists in the Breast Cancer validation dataset. **Table S11** Comparison of performance by subtype of five gene lists in the Ovarian Cancer validation dataset. **Figure S1** A, B, C: Venn diagram of the gene lists from SRF50, 100 and 500 runs for GB, breast cancer and ovarian cancer. **Figure S2**: Histograms of the SRF50, 100 and 500 runs predictions by subtype for GB. **Figure S3**: Histograms of the SRF50, 100 and 500 runs predictions by subtype for breast cancer. **Figure S4**: Histograms of the SRF50, 100 and 500 runs predictions by subtype for ovarian cancer. **Figure S5**: Histograms of the SRF50, Single RF, Verhaak et al, ANOVA and Top 50 ANOVA. **Figure S6** A and B: IPA network plots showing the overlap in the hub genes for the SRF50 gene list and the Verhaak list (grey lines denote the SRF50 gene list and green lines denote the Verhaak gene list). **Figure S7**: Histograms of the SRF50, Single RF, Parker et al, ANOVA and Top 50 ANOVA. **Figure S8** A, B and C: IPA network plots showing the overlap in the hub genes for the SRF50 gene list and the Parker list (grey lines denote the SRF50 gene list and green lines denote the Parker gene list). **Figure S9**: Histograms of the SRF50, Single RF, Tothill et al, ANOVA and Top 50 ANOVA. **Figure S10** A, B, C and D: IPA network plots showing the overlap in the hub genes for the SRF50 gene list and the Tothill list (grey lines denote the SRF50 gene list and green lines denote the Tothill gene list). Click here for file

## References

[B1] Kaiser J Biomarker Tests Need Closer Scrutiny, IOM Concludes Science 2012 335 6076 1554 10.1126/science.335.6076.1554 22461579

[B2] Guyon I Elisseeff A An introduction to variable and feature selection J Mach Learn Res 2003 3 1157 1182

[B3] Saeys Y Inza I Larranaga P A review of feature selection techniques in bioinformatics Bioinformatics 2007 23 19 2507 2517 10.1093/bioinformatics/btm344 17720704

[B4] Tibshirani R Regression Shrinkage and Selection via the Lasso Journal of the Royal Statistical Society Series B (Methodological) 1996 58 1 267 288

[B5] Zou H Hastie T Regression shrinkage and selection via the elastic net, with applications to microarrays 2003 Technical report, Department of Statistics, Stanford University

[B6] Breiman L Random Forests Mach Learn 2001 45 1 5 32 10.1023/A:1010933404324

[B7] Breiman L Friedman JH Olshen R Stone CJ Classification and Regression Tree 1984 Chapman & Hall, Wadsworth, Belmont

[B8] Barnholtz-Sloan JS Guan X Zeigler-Johnson C Meropol NJ Rebbeck TR Decision tree-based modeling of androgen pathway genes and prostate cancer risk Cancer Eπdemiol Biomarkers Prev 2011 20 6 1146 1155 10.1158/1055-9965.EPI-10-0996 PMC3111844 21493872

[B9] Strobl C Zeileis A Brito P Danger: High power! - Exploring the statistical properties of a test for random forest variable importance Proceedings of the 18th International Conference on Computational Statistics: 2008; Porto, Portugal 2008 Physica-Verlag, Heidelberg

[B10] Nicodemus KK Malley JD Strobl C Ziegler A The behaviour of random forest permutation-based variable importance measures under predictor correlation BMC Bioinforma 2010 11 110 10.1186/1471-2105-11-110 PMC2848005 20187966

[B11] Diaz-Uriarte R Alvarez de Andres S Gene selection and classification of microarray data using random forest BMC Bioinforma 2006 7 3 10.1186/1471-2105-7-3 PMC1363357 16398926

[B12] Diaz-Uriarte R GeneSrF and varSelRF: a web-based tool and R package for gene selection and classification using random forest BMC Bioinforma 2007 8 328 10.1186/1471-2105-8-328 PMC2034606 17767709

[B13] Ihaka R Gentleman R R: A Language for Data Analysis and Graphics J Comput Graph Stat 1996 5 3 299 314

[B14] Gentleman R Carey V Bates D Bolstad B Dettling M Dudoit S Ellis B Gautier L Ge Y Gentry J Bioconductor: open software development for computational biology and bioinformatics Genome Biol 2004 5 10 R80 10.1186/gb-2004-5-10-r80 15461798 PMC545600

[B15] Verhaak RG Hoadley KA Purdom E Wang V Qi Y Wilkerson MD Miller CR Ding L Golub T Mesirov JP Integrated genomic analysis identifies clinically relevant subtypes of glioblastoma characterized by abnormalities in PDGFRA, IDH1, EGFR, and NF1 Cancer Cell 2010 17 1 98 110 10.1016/j.ccr.2009.12.020 20129251 PMC2818769

[B16] Tusher VG Tibshirani R Chu G Significance analysis of microarrays applied to the ionizing radiation response Proc Natl Acad Sci U S A 2001 98 9 5116 5121 10.1073/pnas.091062498 11309499 PMC33173

[B17] Dabney AR ClaNC: point-and-click software for classifying microarrays to nearest centroids Bioinformatics 2006 22 1 122 123 10.1093/bioinformatics/bti756 16269418

[B18] Beroukhim R Getz G Nghiemphu L Barretina J Hsueh T Linhart D Vivanco I Lee JC Huang JH Alexander S Assessing the significance of chromosomal aberrations in cancer: methodology and application to glioma Proc Natl Acad Sci U S A 2007 104 50 20007 20012 10.1073/pnas.0710052104 18077431 PMC2148413

[B19] Phillips HS Kharbanda S Chen R Forrest WF Soriano RH Wu TD Misra A Nigro JM Colman H Soroceanu L Molecular subclasses of high-grade glioma predict prognosis, delineate a pattern of disease progression, and resemble stages in neurogenesis Cancer Cell 2006 9 3 157 173 10.1016/j.ccr.2006.02.019 16530701

[B20] Sun L Hui AM Su Q Vortmeyer A Kotliarov Y Pastorino S Passaniti A Menon J Walling J Bailey R Neuronal and glioma-derived stem cell factor induces angiogenesis within the brain Cancer Cell 2006 9 4 287 300 10.1016/j.ccr.2006.03.003 16616334

[B21] Chang HY Nuyten DS Sneddon JB Hastie T Tibshirani R Sorlie T Dai H He YD van’t Veer LJ Bartelink H Robustness, scalability, and integration of a wound-response gene expression signature in predicting breast cancer survival Proc Natl Acad Sci U S A 2005 102 10 3738 3743 10.1073/pnas.0409462102 15701700 PMC548329

[B22] Parker JS Mullins M Cheang MC Leung S Voduc D Vickery T Davies S Fauron C He X Hu Z Supervised risk predictor of breast cancer based on intrinsic subtypes J Clin Oncol 2009 27 8 1160 1167 10.1200/JCO.2008.18.1370 19204204 PMC2667820

[B23] Sørlie T Perou CM Tibshirani R Aas T Geisler S Johnsen H Hastie T Eisen MB van de Rijn M Jeffrey SS Gene expression patterns of breast carcinomas distinguish tumor subclasses with clinical implications Proc Natl Acad Sci 2001 98 19 10869 10874 10.1073/pnas.191367098 11553815 PMC58566

[B24] Hu Z Fan C Oh D Marron J He X Qaqish B Livasy C Carey L Reynolds E Dressler L The molecular portraits of breast tumors are conserved across microarray platforms BMC Genomics 2006 7 1 96 10.1186/1471-2164-7-96 16643655 PMC1468408

[B25] Perreard L Fan C Quackenbush JF Mullins M Gauthier NP Nelson E Mone M Hansen H Buys SS Rasmussen K Classification and risk stratification of invasive breast carcinomas using a real-time quantitative RT-PCR assay Breast Cancer Res 2006 8 2 R23 10.1186/bcr1399 16626501 PMC1557722

[B26] Sørlie T Tibshirani R Parker J Hastie T Marron JS Nobel A Deng S Johnsen H Pesich R Geisler S Repeated observation of breast tumor subtypes in independent gene expression data sets Proc Natl Acad Sci 2003 100 14 8418 8423 10.1073/pnas.0932692100 12829800 PMC166244

[B27] Tothill RW Tinker AV George J Brown R Fox SB Lade S Johnson DS Trivett MK Etemadmoghadam D Locandro B Novel molecular subtypes of serous and endometrioid ovarian cancer linked to clinical outcome Clin Cancer Res 2008 14 16 5198 5208 10.1158/1078-0432.CCR-08-0196 18698038

[B28] Reiner A Yekutieli D Benjamini Y Identifying differentially expressed genes using false discovery rate controlling procedures Bioinformatics 2003 19 3 368 375 10.1093/bioinformatics/btf877 12584122

[B29] Hassan MR Ramamohanarao K Karmakar C Hossain MM Bailey J Zaki MJ, Yu JX, Ravindran B, Pudi V A novel scalable multi-class ROC for effective visualization and computation Proceedings of the 14th Pacific-Asia conference on Advances in Knowledge Discovery and Data Mining - Volume Part I 2010 Springer-Verlag, Hyderabad, India

[B30] Kleihues P Ohgaki H Genetics of Glioma Progression and the Definition of Primary and Secondary Glioblastoma Brain Pathology 1997 7 4 1131 1136 10.1111/j.1750-3639.1997.tb00993.x

[B31] Kleihues P Ohgaki H Primary and secondary glioblastomas: from concept to clinical diagnosis Neuro Oncol 1999 1 1 44 51 11550301 10.1093/neuonc/1.1.44 PMC1919466

[B32] Ohgaki H Dessen P Jourde B Horstmann S Nishikawa T Di Patre PL Burkhard C Schuler D Probst-Hensch NM Maiorka PC Genetic pathways to glioblastoma: a population-based study Cancer Res 2004 64 19 6892 6899 10.1158/0008-5472.CAN-04-1337 15466178

[B33] Ohgaki H Kleihues P Eπdemiology and etiology of gliomas Acta Neuropathol 2005 109 1 93 108 10.1007/s00401-005-0991-y 15685439

[B34] Ishwaran H Kogalur UB Chen X Minn AJ Random survival forests for high-dimensional data Statistical Analysis and Data Mining 2011 4 1 115 132 10.1002/sam.10103

