# Supplementary material for: Splitting random forest (SRF) for determining compact sets of genes that distinguish between cancer subtypes
Source: J Clin Bioinforma. 2012 May 22;2:13. doi: 10.1186/2043-9113-2-13 (PMC3444418; doi:10.1186/2043-9113-2-13)
Supplement: Additional file 1 — Table S1 Comparison of performance by subtype of SRF50, 100 and 500 runs in the GB full dataset. Table S2 Comparison of performance by subtype of SRF50, 100 and 500 runs in the breast cancer training dataset. Table S3 Comparison of performance by subtype of SRF50, 100 and 500 runs in the ovarian cancer training dataset. Table S4 Comparison of the gene lists from SRF50, 100 and 500 runs for GB. Table S5 Comparisons of the gene lists from SRF50, 100 and 500 runs for breast cancer. Table S6 Comparisons of the gene lists from SRF50, 100 and 500 runs for ovarian cancer. Table S7 Comparison of performance by subtype of five gene lists in the GB validation dataset. Table S8 Pairwise AUC comparison between subtypes of five gene lists in the GB, BC and OC validation dataset. Table S9 Hub genes in the top networks from IPA analysis for the SRF50 and published gene lists for GB, breast cancer (BC) and ovarian cancer (OC). Table S10 Comparison of performance by subtype of five gene lists in the Breast Cancer validation dataset. Table S11 Comparison of performance by subtype of five gene lists in the Ovarian Cancer validation dataset. Figure S1 A, B, C: Venn diagram of the gene lists from SRF50, 100 and 500 runs for GB, breast cancer and ovarian cancer. Figure S2: Histograms of the SRF50, 100 and 500 runs predictions by subtype for GB. Figure S3: Histograms of the SRF50, 100 and 500 runs predictions by subtype for breast cancer. Figure S4: Histograms of the SRF50, 100 and 500 runs predictions by subtype for ovarian cancer. Figure S5: Histograms of the SRF50, Single RF, Verhaak et al, ANOVA and Top 50 ANOVA. Figure S6 A and B: IPA network plots showing the overlap in the hub genes for the SRF50 gene list and the Verhaak list (grey lines denote the SRF50 gene list and green lines denote the Verhaak gene list). Figure S7: Histograms of the SRF50, Single RF, Parker et al, ANOVA and Top 50 ANOVA. Figure S8 A, B and C: IPA network plots showing the overlap in the hub genes for the SRF [file 2043-9113-2-13-S1.doc]

**Supplementary table 1: Comparison of performance by subtype of SRF50, 100 and 500 runs in the GB** full dataset

| **SRF50 (Overall Prediction Accuracy=95.4%)** | **Classical (N=19)** | **Mesenchymal(N=18)** | **Neural(N=13)** | **Proneural(N=27)** |
| --- | --- | --- | --- | --- |
| **Classical (N=19)** | 17 (89%) | 0 (0%) | 1 (5%) | 1 (5%) |
| **Mesenchymal (N=18)** | 1 (4%) | 27 (96%) | 0 (0%) | 0 (0%) |
| **Neural (N=13)** | 0 (0%) | 1 (8%) | 12 (92%) | 0 (0%) |
| **Proneural (N=27)** | 0 (0%) | 0 (0%) | 0 (0%) | 27 (100%) |
| **SRF100 (Overall Prediction Accuracy=96.6%)** | **Classical (N=19)** | **Mesenchymal(N=18)** | **Neural(N=13)** | **Proneural(N=27)** |
| **Classical (N=19)** | 18 (95%) | 1 (5%) | 0 (0%) | 0 (0%) |
| **Mesenchymal (N=18)** | 1 (4%) | 27 (96%) | 0 (0%) | 0 (0%) |
| **Neural (N=13)** | 0 (0%) | 1 (8%) | 12 (92%) | 0 (0%) |
| **Proneural (N=27)** | 0 (0%) | 0 (0%) | 0 (0%) | 27 (100%) |
| **SRF500 (Overall Prediction Accuracy=98.9%)** | **Classical (N=19)** | **Mesenchymal(N=18)** | **Neural(N=13)** | **Proneural(N=27)** |
| **Classical (N=19)** | 19 (100%) | 0 (0%) | 0 (0%) | 0 (0%) |
| **Mesenchymal (N=18)** | 0 (0%) | 28 (100%) | 0 (0%) | 0 (0%) |
| **Neural (N=13)** | 0 (0%) | 1 (8%) | 12 (92%) | 0 (0%) |
| **Proneural (N=27)** | 0 (0%) | 0 (0%) | 0 (0%) | 27 (100%) |

*Fisher’s EXACT test P-value for the SRF 50, 100 and 500 runs comparison is 0.9984.

**Supplementary table 2: Comparison of performance by subtype of SRF50, 100 and 500 runs in the breast cancer** training dataset

| **SRF50 (Overall Prediction Accuracy=93.6%)** | **Basal-Like (N=15)** | **HER2 (N=5)** | **Luminal A (N=11)** | **Luminal B (N=16)** |
| --- | --- | --- | --- | --- |
| **Basal-Like (N=15)** | 15 (100%) | 0 (0%) | 0 (0%) | 0 (0%) |
| **HER2 (N=5)** | 1 (20%) | 4 (80%) | 0 (0%) | 0 (0%) |
| **Luminal A (N=11)** | 0 (0%) | 0 (0%) | 10 (91%) | 1 (9%) |
| **Luminal B (N=16)** | 0 (0%) | 0 (0%) | 1 (6%) | 15 (94%) |
| **SRF100 (Overall Prediction Accuracy=93.6%)** | **Basal-Like (N=15)** | **HER2 (N=5)** | **Luminal A (N=11)** | **Luminal B (N=16)** |
| **Basal-Like (N=15)** | 15 (100%) | 0 (0%) | 0 (0%) | 0 (0%) |
| **HER2 (N=5)** | 0 (0%) | 4 (80%) | 1 (20%) | 0 (0%) |
| **Luminal A (N=11)** | 0 (0%) | 0 (0%) | 10 (91%) | 1 (9%) |
| **Luminal B (N=16)** | 0 (0%) | 0 (0%) | 1 (6%) | 15 (94%) |
| **SRF500 (Overall Prediction Accuracy=95.7%)** | **Basal-Like (N=15)** | **HER2 (N=5)** | **Luminal A (N=11)** | **Luminal B (N=16)** |
| **Basal-Like (N=15)** | 15 (100%) | 0 (0%) | 0 (0%) | 0 (0%) |
| **HER2 (N=5)** | 0 (0%) | 4 (80%) | 1 (20%) | 0 (0%) |
| **Luminal A (N=11)** | 0 (0%) | 1 (9%) | 10 (91%) | 0 (0%) |
| **Luminal B (N=16)** | 0 (0%) | 0 (0%) | 0 (0%) | 16 (100%) |

*Fisher’s EXACT test P-value for the SRF 50, 100 and 500 runs comparison is 0.9999.

**Supplementary table 3: Comparison of performance by subtype of SRF50, 100 and 500 runs in the ovarian cancer training dataset**

| **SRF50 (Overall Prediction Accuracy=92.7%)** | **C1 (N=21)** | **C2 (N=13)** | **C4 (N=12)** | **C5 (N=9)** |
| --- | --- | --- | --- | --- |
| **C1 (N=21)** | 20 (95%) | 1 (5%) | 0 (0%) | 0 (0%) |
| **C2 (N=13)** | 1 (8%) | 12 (92%) | 0 (0%) | 0 (0%) |
| **C4 (N=12)** | 0 (0%) | 0 (0%) | 12 (100%) | 0 (0%) |
| **C5 (N=9)** | 0 (0%) | 1 (11%) | 1 (11%) | 7 (78%) |
| **SRF100 (Overall Prediction Accuracy=94.5%)** | **C1 (N=21)** | **C2 (N=13)** | **C4 (N=12)** | **C5 (N=9)** |
| **C1 (N=21)** | 21 (100%) | 0 (0%) | 0 (0%) | 0 (0%) |
| **C2 (N=13)** | 0 (0%) | 12 (92%) | 1 (8%) | 0 (0%) |
| **C4 (N=12)** | 0 (0%) | 0 (0%) | 12 (100%) | 0 (0%) |
| **C5 (N=9)** | 1 (11%) | 0 (0%) | 1 (11%) | 7 (78%) |
| **SRF500 (Overall Prediction Accuracy=94.5%)** | **C1 (N=21)** | **C2 (N=13)** | **C4 (N=12)** | **C5 (N=9)** |
| **C1 (N=21)** | 20 (95%) | 0 (0%) | 1 (5%) | 0 (0%) |
| **C2 (N=13)** | 0 (0%) | 13 (100%) | 0 (0%) | 0 (0%) |
| **C4 (N=12)** | 0 (0%) | 2 (17%) | 10 (83%) | 0 (0%) |
| **C5 (N=9)** | 0 (0%) | 0 (0%) | 0 (0%) | 9 (100%) |

*Fisher’s EXACT test P-value for the SRF 50, 100 and 500 runs comparison is 0.4057.

**Supplementary table 4: Comparisons of the gene lists from SRF50, 100 and 500 runs for** GB

| **SRF50 (N=36)** | | **SRF 100 (N=23)** | | **SRF500 (N=29)** | | **Overlap 50 VS 100 (N=11)** | **Overlap of 50 VS 500 (N=14)** | **Overlap of 100 VS 500 (N=13)** | **Overlap of the 3 (N=9)** |
| --- | --- | --- | --- | --- | --- | --- | --- | --- | --- |
| KLRC4 | BNC2 | DCX | ANKRD25 | KLRC4 | MRC2 | BCL7A | BCL7A | AMOTL2 | BCL7A |
| FMNL1 | RAC2 | KLRC4 | VDR | AMOTL2 | KLRC3 | CHD7 | CHD7 | CSPG5 | CHD7 |
| FER1L3 | DCBLD2 | AMOTL2 | KLRC3 | PAK7 | SHC1 | EPHB1 | EPHB1 | KIF21B | EPHB1 |
| KIAA1166 | MRC2 | FER1L3 | EPHB1 | FER1L3 | EPHB1 | FAM77C | FAM77C | RAB33A | FAM77C |
| FAM77C | CD97 | KIAA1166 | RAB11FIP1 | RBMS1 | FHOD3 | FER1L3 | FER1L3 | BCL7A | FER1L3 |
| WASF1 | VDR | IQGAP1 |  | KIAA1166 | OLIG2 | KIAA1166 | FHOD3 | CHD7 | KIAA1166 |
| RBPJ | KLRC3 | FAM77C |  | TGFBR2 | EPHB4 | KLRC3 | IL4R | EPHB1 | KLRC3 |
| CTSB | ALCAM | KIF21B |  | FAM77C | C6orf134 | KLRC4 | KIAA1166 | FAM77C | KLRC4 |
| NOL4 | SCN3A | WASF1 |  | KIF21B | MYT1 | RAB11FIP1 | KLRC3 | FER1L3 | WASF1 |
| IL4R | FLJ21963 | PPM1E |  | WASF1 | CLASP2 | VDR | KLRC4 | KIAA1166 |  |
| PLAUR | CLIC1 | LILRB3 |  | FXYD6 | DNM3 | WASF1 | MRC2 | KLRC3 |  |
| ZNF217 | EPHB1 | CSPG5 |  | CSPG5 |  |  | WASF1 | KLRC4 |  |
| MAN2A1 | FHOD3 | CRMP1 |  | IL4R |  |  |  | WASF1 |  |
| ITGA5 | ARPC1B | RAB33A |  | MLLT11 |  |  |  |  |  |
| BCL7A | RAB11FIP1 | KYNU |  | RAB33A |  |  |  |  |  |
| ELF4 | PFN2 | TCIRG1 |  | RP11_35N6_1 |  |  |  |  |  |
| CHD7 | CXXC4 | BCL7A |  | BCL7A |  |  |  |  |  |
| SLC10A3 | RAB27A | CHD7 |  | CHD7 |  |  |  |  |  |

**Supplementary table 5: Comparisons of the gene lists from SRF50, 100 and 500 runs for** breast cancer

| **50 runs (N=48)** | | **100 runs (N=50)** | | **500 runs (N=32)** | | **50 VS 100 (N=23)** | **50 VS 500 (N=9)** | **100VS 500 (N=10)** | **Overlap of the 3 (N=9)** |
| --- | --- | --- | --- | --- | --- | --- | --- | --- | --- |
| ACADSB | KIAA0062 | ADORA2B | FOXC1 | APRT | NFE2L3 | AGR2 | CGI-52 | CAPN2 | CGI-52 |
| ADCY1 | KIAA0575 | AGR2 | FXYD5 | CAPN2 | NPDC1 | CA12 | COQ7 | CGI-52 | COQ7 |
| AGR2 | KIAA0882 | BENE | FZD9 | CGI-52 | PHGDH | COX6C | DKFZP434I114 | COQ7 | DKFZP434I114 |
| ARHI | KIAA1209 | C4A | GATA3 | COQ7 | PIM1 | DKFZp762A227 | FOXC1 | DKFZP434I114 | FOXC1 |
| BCAS1 | KIF13B | CA12 | HMGIY | COX17 | PTPRK | ESR1 | GATA3 | FOXC1 | GATA3 |
| CA12 | MAPRE2 | CAPN2 | HNF3A | D123 | SLC1A1 | FBP1 | HNF3A | GATA3 | HNF3A |
| CGI-52 | MSN | CDC20 | JCL-1 | DKFZP434I114 | UGCG | FLJ10980 | KIAA0882 | HNF3A | KIAA0882 |
| COQ7 | MTL5 | CDC25B | KIAA0575 | DKFZP586G1517 |  | FLJ20273 | MSN | KIAA0882 | MSN |
| COX6C | MYB | CDC4L | KIAA0882 | DSC2 |  | JCL-1 | PIM1 | MSN | PIM1 |
| DKFZP434I114 | NET-6 | CDKN2A | KIAA1278 | FLJ10173 |  | KIAA0575 |  | PIM1 |  |
| DKFZp762A227 | P28 | CG1I | KIAA1467 | FLJ10659 |  | NET-6 |  |  |  |
| DYSF | PGM1 | CGI-52 | MFGE8 | FLJ10996 |  | PGM1 |  |  |  |
| ESR1 | PIM1 | CHST2 | MSN | FLJ23293 |  | PRO1659 |  |  |  |
| FBP1 | PRO1659 | COQ7 | NET-6 | FOXC1 |  | XBP1 |  |  |  |
| FLJ10116 | PRRG2 | COX6C | NME1 | G2 |  | CGI-52 |  |  |  |
| FLJ10724 | RBMS1 | DKFZP434F124 | PGM1 | GABRP |  | COQ7 |  |  |  |
| FLJ10980 | SEC15L | DKFZP434I114 | PIM1 | GATA3 |  | DKFZP434I114 |  |  |  |
| FLJ20262 | SLC22A5 | DKFZp762A227 | PLCG2 | GNAI1 |  | FOXC1 |  |  |  |
| FLJ20273 | SSR2 | ESR1 | PLD1 | HNF3A |  | GATA3 |  |  |  |
| FOXC1 | TJP3 | ETS2 | PRO1659 | JDP1 |  | HNF3A |  |  |  |
| GATA3 | TOM1L1 | FBP1 | RARA | KIAA0882 |  | KIAA0882 |  |  |  |
| HMGCL | WBP1 | FLJ10980 | SLC16A6 | KIAA1013 |  | MSN |  |  |  |
| HNF3A | XBP1 | FLJ20273 | SPS2 | MPB1 |  | PIM1 |  |  |  |
| HYPK |  | FLJ20499 | TBX3-iso | MSN |  |  |  |  |  |
| JCL-1 |  | FLJ20515 | XBP1 | MTMR2 |  |  |  |  |  |

**Supplementary table 6: Comparisons of the gene lists from SRF50, 100 and 500 runs for** ovarian cancer

| **50 runs (N=189)** | | **100 runs (N=290)** | | | **500 runs (N=188)** | | **50 VS 100 (N=120)** | **50 VS 500 (N=107)** | **100VS 500 (N=118)** | **Overlap of the 3 (N=81)** |
| --- | --- | --- | --- | --- | --- | --- | --- | --- | --- | --- |
| BMP1 | CCL11 | ANGPT1 | ANTXR2 | MATN3 | ACTA2 | CD53 | ANTXR2 | ASPN | ANGPT1 | ACVRL1 |
| C1QTNF2 | CD53 | ARHGAP15 | ANXA5 | MMP19 | ALPK2 | CD84 | ANXA5 | BHLHE22 | ARHGAP15 | ADAM12 |
| CD14 | CD84 | CDH11 | ASAM | MRAS | ANXA11 | COL1A1 | ASAM | C3AR1 | CDH11 | ADAMTS12 |
| CD248 | COL1A1 | CHST11 | BACH2 | MRGPRF | AOAH | COL1A2 | BACH2 | CLEC4A | CHST11 | ADAMTSL1 |
| CREB3L1 | COL1A2 | CILP | CD163 | MRVI1 | BEND6 | COL3A1 | CD163 | COL10A1 | CILP | AEBP1 |
| DACT3 | COL3A1 | COLEC12 | CD86 | MS4A4A | BICC1 | COL5A1 | CD86 | COL11A1 | COLEC12 | ANGPTL2 |
| DOK3 | COL5A1 | DCN | CILP2 | MXRA8 | C10orf79 | COL5A2 | CILP2 | COPZ2 | DCN | ANTXR1 |
| ECM2 | COL5A2 | DNMT3A | COL6A2 | NCF2 | C1orf162 | COL6A1 | COL6A2 | CTSK | DNMT3A | BGN |
| EMILIN1 | COL6A1 | DOCK2 | CTHRC1 | NID1 | C7orf10 | COL6A3 | CTHRC1 | GIMAP7 | DOCK2 | C1QTNF5 |
| FCGR2B | COL6A3 | EVI2A | CTSS | NKG7 | CD300A | CRISPLD2 | CTSS | GIMAP8 | EVI2A | C1QTNF6 |
| FERMT3 | CRISPLD2 | EVI2B | EDNRA | NUP43 | CEACAM6 | CXCL14 | EDNRA | GUCY1A3 | EVI2B | CACNA1C |
| FKBP7 | CXCL14 | GAS7 | EMP3 | OSBPL5 | CSF1R | ECM1 | EMP3 | HSD17B6 | GAS7 | CCDC80 |
| FNIP2 | ECM1 | GIMAP1 | FAM155A | P4HA3 | CSMD2 | ENTPD1 | FAM155A | INHBA | GIMAP1 | CCL11 |
| GPR183 | ENTPD1 | GLRX | FCGR1B | PARVA | CXCR7 | EPB41L3 | FCGR1B | ITGA4 | GLRX | CD53 |
| ITGBL1 | EPB41L3 | GPR65 | FNDC1 | PDLIM3 | DIP2C | FBLN1 | FNDC1 | LOC100128821 | GPR65 | CD84 |
| KIAA1949 | FBLN1 | HLX | GALNT10 | PDZRN3 | DNM3 | FBLN2 | GALNT10 | LRRC15 | HLX | COL1A1 |
| LCP2 | FBLN2 | ISM1 | GFPT2 | PHF23 | FAP | FBN1 | GFPT2 | MFAP4 | ISM1 | COL1A2 |
| LHFPL2 | FBN1 | KCNE4 | GIMAP6 | PHLDB1 | GRP | FCGR1A | GIMAP6 | MMP11 | KCNE4 | COL3A1 |
| LOC145786 | FCGR1A | LOC401097 | GLI3 | PIP4K2A | IL13RA1 | FN1 | GLI3 | MNDA | LOC401097 | COL5A1 |
| LOC149134 | FN1 | LUM | GREM1 | PLEK | ITGA11 | FPR3 | GREM1 | ODZ3 | LUM | COL5A2 |
| MAPRE2 | FPR3 | MOXD1 | LAPTM5 | PTGER4 | LAT2 | FRMD6 | LAPTM5 | PAPPA | MOXD1 | COL6A1 |
| MEIS3 | FRMD6 | MS4A6A | LY96 | PTGIS | LTBP2 | FSTL1 | LY96 | SMOC2 | MS4A6A | COL6A3 |
| MEOX2 | FSTL1 | MS4A7 | LZTS1 | RAP2B | LY86 | FZD1 | LZTS1 | TBXAS1 | MS4A7 | CRISPLD2 |
| MLLT11 | FZD1 | MSRB3 | MAFB | RCAN1 | MLX | GLIPR1 | MAFB | TIMP3 | MSRB3 | CXCL14 |
| MN1 | GLIPR1 | NALCN | MARCKS | RGS16 | MPEG1 | GLT8D2 | MARCKS | TNFAIP6 | NALCN | ECM1 |
| MYO1B | GLT8D2 | NID2 | NPL | RGS18 | MYCN | GMFG | NPL | TNFSF13B | NID2 | ENTPD1 |
| 1-Mar | GMFG | NNMT | OLFML3 | RNASE4 | MYO1F | GPRIN3 | OLFML3 | ACVRL1 | NNMT | EPB41L3 |
| NKD2 | GPRIN3 | PLXDC1 | PALLD | SERTAD2 | NBL1 | HNT | PALLD | ADAM12 | PLXDC1 | FBLN1 |
| NOD2 | HNT | PLXND1 | PECAM1 | SFXN3 | NKX3-2 | IFFO1 | PECAM1 | ADAMTS12 | PLXND1 | FBLN2 |
| PPAP2A | IFFO1 | PMP22 | PLAU | SH3RF3 | NLRP3 | KIF26B | PLAU | ADAMTSL1 | PMP22 | FBN1 |
| PRICKLE1 | KIF26B | POSTN | RCN3 | SLC12A8 | PLK2 | LAIR1 | RCN3 | AEBP1 | POSTN | FCGR1A |
| PTPRD | LAIR1 | PTPRC | RUNX1T1 | SLC7A7 | RNFT2 | LAMB1 | RUNX1T1 | ANGPTL2 | PTPRC | FN1 |
| RECK | LAMB1 | SASH3 | SAMSN1 | SLC9A9 | RUNX1 | LHFP | SAMSN1 | ANTXR1 | SASH3 | FPR3 |
| RTP4 | LHFP | SULF1 | SH3PXD2B | SLIT3 | SEMA3C | LOC26010 | SH3PXD2B | BGN | SULF1 | FRMD6 |
| SLA | LOC26010 | TGFBR2 | SLAMF8 | STK10 | SNN | LST1 | SLAMF8 | C1QTNF5 | TGFBR2 | FSTL1 |
| SMPDL3A | LST1 | ZCCHC24 | SPOCK1 | SULF2 | SPAG9 | MAP4K4 | SPOCK1 | C1QTNF6 | ZCCHC24 | FZD1 |
| SOAT1 | MAP4K4 | ZEB1 | SRGN | TARP | STX7 | MMP2 | SRGN | CACNA1C | ZEB1 | GLIPR1 |
| ST8SIA4 | MMP2 | ABL1 | TIMP2 | TCF4 | 11-Sep | MST150 | TIMP2 | CCDC80 | ACVRL1 | GLT8D2 |
| SYDE1 | MST150 | ACSL5 | XYLT1 | TCF7L1 | TANC2 | NAGA | XYLT1 | CCL11 | ADAM12 | GMFG |
| 8-Sep | NAGA | AGTR1 | ACVRL1 | TGFB3 | TFEC | NT5E | ACVRL1 | CD53 | ADAMTS12 | GPRIN3 |
| TGFBR1 | NT5E | ALDH1A3 | ADAM12 | TMEM200A | TLR7 | NUAK1 | ADAM12 | CD84 | ADAMTSL1 | HNT |
| TMEM119 | NUAK1 | AMOTL1 | ADAMTS12 | TP53INP2 | TMEM45A | OLFML2B | ADAMTS12 | COL1A1 | AEBP1 | IFFO1 |
| TNC | OLFML2B | ARFGAP3 | ADAMTSL1 | TRAM2 | TYROBP | PDGFRB | ADAMTSL1 | COL1A2 | ANGPTL2 | KIF26B |
| ASPN | PDGFRB | ARHGAP18 | AEBP1 | TSPAN18 | WISP1 | PMEPA1 | AEBP1 | COL3A1 | ANTXR1 | LAIR1 |
| BHLHE22 | PMEPA1 | ARHGAP28 | ANGPTL2 | VAT1 | ANGPT1 | PRKG1 | ANGPTL2 | COL5A1 | BGN | LAMB1 |
| C3AR1 | PRKG1 | ARSB | ANTXR1 | VCAM1 | ARHGAP15 | PRRX1 | ANTXR1 | COL5A2 | C1QTNF5 | LHFP |
| CLEC4A | PRRX1 | C18orf10 | BGN | WT1 | CDH11 | PTGER3 | BGN | COL6A1 | C1QTNF6 | LOC26010 |
| COL10A1 | PTGER3 | C1orf114 | C1QTNF5 | YPEL5 | CHST11 | PTRF | C1QTNF5 | COL6A3 | CACNA1C | LST1 |
| COL11A1 | PTRF | C6orf190 | C1QTNF6 | ZNF469 | CILP | RAB31 | C1QTNF6 | CRISPLD2 | CCDC80 | MAP4K4 |
| COPZ2 | RAB31 | CALD1 | CACNA1C | ZNF667 | COLEC12 | RCSD1 | CACNA1C | CXCL14 | CCL11 | MMP2 |
| CTSK | RCSD1 | CCL5 | CCDC80 |  | DCN | SERPINF1 | CCDC80 | ECM1 | CD53 | MST150 |
| GIMAP7 | SERPINF1 | CCR1 | CCL11 |  | DNMT3A | SFRP2 | CCL11 | ENTPD1 | CD84 | NAGA |
| GIMAP8 | SFRP2 | CCR7 | CD53 |  | DOCK2 | SNAI2 | CD53 | EPB41L3 | COL1A1 | NT5E |
| GUCY1A3 | SNAI2 | CD37 | CD84 |  | EVI2A | SNX10 | CD84 | FBLN1 | COL1A2 | NUAK1 |
| HSD17B6 | SNX10 | CD48 | COL1A1 |  | EVI2B | SPARC | COL1A1 | FBLN2 | COL3A1 | OLFML2B |
| INHBA | SPARC | CD52 | COL1A2 |  | GAS7 | SPHK1 | COL1A2 | FBN1 | COL5A1 | PDGFRB |
| ITGA4 | SPHK1 | CD93 | COL3A1 |  | GIMAP1 | SPON2 | COL3A1 | FCGR1A | COL5A2 | PMEPA1 |
| LOC100128821 | SPON2 | CDGAP | COL5A1 |  | GLRX | SRPX | COL5A1 | FN1 | COL6A1 | PRKG1 |
| LRRC15 | SRPX | CERCAM | COL5A2 |  | GPR65 | SYTL2 | COL5A2 | FPR3 | COL6A3 | PRRX1 |
| MFAP4 | SYTL2 | CIITA | COL6A1 |  | HLX | TGFBI | COL6A1 | FRMD6 | CRISPLD2 | PTGER3 |
| MMP11 | TGFBI | CLEC2B | COL6A3 |  | ISM1 | THBS1 | COL6A3 | FSTL1 | CXCL14 | PTRF |
| MNDA | THBS1 | CLEC7A | CRISPLD2 |  | KCNE4 | THBS2 | CRISPLD2 | FZD1 | ECM1 | RAB31 |
| ODZ3 | THBS2 | CNN2 | CXCL14 |  | LOC401097 | TICAM2 | CXCL14 | GLIPR1 | ENTPD1 | RCSD1 |
| PAPPA | TICAM2 | COL8A2 | ECM1 |  | LUM | TLR4 | ECM1 | GLT8D2 | EPB41L3 | SERPINF1 |
| SMOC2 | TLR4 | CORO1C | ENTPD1 |  | MOXD1 | TMEM158 | ENTPD1 | GMFG | FBLN1 | SFRP2 |
| TBXAS1 | TMEM158 | CXCL17 | EPB41L3 |  | MS4A6A | VCAN | EPB41L3 | GPRIN3 | FBLN2 | SNAI2 |
| TIMP3 | VCAN | CYR61 | FBLN1 |  | MS4A7 | WIPF1 | FBLN1 | HNT | FBN1 | SNX10 |
| TNFAIP6 | WIPF1 | DCHS1 | FBLN2 |  | MSRB3 | ZEB2 | FBLN2 | IFFO1 | FCGR1A | SPARC |
| TNFSF13B | ZEB2 | DLL1 | FBN1 |  | NALCN |  | FBN1 | KIF26B | FN1 | SPHK1 |
| ANTXR2 |  | DOCK8 | FCGR1A |  | NID2 |  | FCGR1A | LAIR1 | FPR3 | SPON2 |
| ANXA5 |  | EMCN | FN1 |  | NNMT |  | FN1 | LAMB1 | FRMD6 | SRPX |
| ASAM |  | EMR2 | FPR3 |  | PLXDC1 |  | FPR3 | LHFP | FSTL1 | SYTL2 |
| BACH2 |  | ENTPD4 | FRMD6 |  | PLXND1 |  | FRMD6 | LOC26010 | FZD1 | TGFBI |
| CD163 |  | ETS1 | FSTL1 |  | PMP22 |  | FSTL1 | LST1 | GLIPR1 | THBS1 |
| CD86 |  | ETV1 | FZD1 |  | POSTN |  | FZD1 | MAP4K4 | GLT8D2 | THBS2 |
| CILP2 |  | FAM101B | GLIPR1 |  | PTPRC |  | GLIPR1 | MMP2 | GMFG | TICAM2 |
| COL6A2 |  | FAM38B | GLT8D2 |  | SASH3 |  | GLT8D2 | MST150 | GPRIN3 | TLR4 |
| CTHRC1 |  | FAM65A | GMFG |  | SULF1 |  | GMFG | NAGA | HNT | TMEM158 |
| CTSS |  | FCER1G | GPRIN3 |  | TGFBR2 |  | GPRIN3 | NT5E | IFFO1 | VCAN |
| EDNRA |  | FLI1 | HNT |  | ZCCHC24 |  | HNT | NUAK1 | KIF26B | WIPF1 |
| EMP3 |  | FSTL3 | IFFO1 |  | ZEB1 |  | IFFO1 | OLFML2B | LAIR1 | ZEB2 |
| FAM155A |  | FYB | KIF26B |  | ASPN |  | KIF26B | PDGFRB | LAMB1 |  |
| FCGR1B |  | GALNT1 | LAIR1 |  | BHLHE22 |  | LAIR1 | PMEPA1 | LHFP |  |
| FNDC1 |  | GALNTL2 | LAMB1 |  | C3AR1 |  | LAMB1 | PRKG1 | LOC26010 |  |
| GALNT10 |  | GEM | LHFP |  | CLEC4A |  | LHFP | PRRX1 | LST1 |  |
| GFPT2 |  | GIMAP5 | LOC26010 |  | COL10A1 |  | LOC26010 | PTGER3 | MAP4K4 |  |
| GIMAP6 |  | GJB2 | LST1 |  | COL11A1 |  | LST1 | PTRF | MMP2 |  |
| GLI3 |  | GJC1 | MAP4K4 |  | COPZ2 |  | MAP4K4 | RAB31 | MST150 |  |
| GREM1 |  | GNG2 | MMP2 |  | CTSK |  | MMP2 | RCSD1 | NAGA |  |
| LAPTM5 |  | GPC6 | MST150 |  | GIMAP7 |  | MST150 | SERPINF1 | NT5E |  |
| LY96 |  | GPNMB | NAGA |  | GIMAP8 |  | NAGA | SFRP2 | NUAK1 |  |
| LZTS1 |  | GZMK | NT5E |  | GUCY1A3 |  | NT5E | SNAI2 | OLFML2B |  |
| MAFB |  | HHIP | NUAK1 |  | HSD17B6 |  | NUAK1 | SNX10 | PDGFRB |  |
| MARCKS |  | HOMER3 | OLFML2B |  | INHBA |  | OLFML2B | SPARC | PMEPA1 |  |
| NPL |  | IFI30 | PDGFRB |  | ITGA4 |  | PDGFRB | SPHK1 | PRKG1 |  |
| OLFML3 |  | IGF2R | PMEPA1 |  | LOC100128821 |  | PMEPA1 | SPON2 | PRRX1 |  |
| PALLD |  | IGFBP4 | PRKG1 |  | LRRC15 |  | PRKG1 | SRPX | PTGER3 |  |
| PECAM1 |  | IGH | PRRX1 |  | MFAP4 |  | PRRX1 | SYTL2 | PTRF |  |
| PLAU |  | IGHM | PTGER3 |  | MMP11 |  | PTGER3 | TGFBI | RAB31 |  |
| RCN3 |  | IGKC | PTRF |  | MNDA |  | PTRF | THBS1 | RCSD1 |  |
| RUNX1T1 |  | IRF8 | RAB31 |  | ODZ3 |  | RAB31 | THBS2 | SERPINF1 |  |
| SAMSN1 |  | ISLR | RCSD1 |  | PAPPA |  | RCSD1 | TICAM2 | SFRP2 |  |
| SH3PXD2B |  | JAM3 | SERPINF1 |  | SMOC2 |  | SERPINF1 | TLR4 | SNAI2 |  |
| SLAMF8 |  | KLF2 | SFRP2 |  | TBXAS1 |  | SFRP2 | TMEM158 | SNX10 |  |
| SPOCK1 |  | KLF6 | SNAI2 |  | TIMP3 |  | SNAI2 | VCAN | SPARC |  |
| SRGN |  | KRI1 | SNX10 |  | TNFAIP6 |  | SNX10 | WIPF1 | SPHK1 |  |
| TIMP2 |  | LAMA4 | SPARC |  | TNFSF13B |  | SPARC | ZEB2 | SPON2 |  |
| XYLT1 |  | LEPRE1 | SPHK1 |  | ACVRL1 |  | SPHK1 |  | SRPX |  |
| ACVRL1 |  | LEPREL2 | SPON2 |  | ADAM12 |  | SPON2 |  | SYTL2 |  |
| ADAM12 |  | LILRB1 | SRPX |  | ADAMTS12 |  | SRPX |  | TGFBI |  |
| ADAMTS12 |  | LILRB4 | SYTL2 |  | ADAMTSL1 |  | SYTL2 |  | THBS1 |  |
| ADAMTSL1 |  | LIMA1 | TGFBI |  | AEBP1 |  | TGFBI |  | THBS2 |  |
| AEBP1 |  | LOC283551 | THBS1 |  | ANGPTL2 |  | THBS1 |  | TICAM2 |  |
| ANGPTL2 |  | LOC441461 | THBS2 |  | ANTXR1 |  | THBS2 |  | TLR4 |  |
| ANTXR1 |  | LOC644242 | TICAM2 |  | BGN |  | TICAM2 |  | TMEM158 |  |
| BGN |  | LOX | TLR4 |  | C1QTNF5 |  | TLR4 |  | VCAN |  |
| C1QTNF5 |  | LOXL1 | TMEM158 |  | C1QTNF6 |  | TMEM158 |  | WIPF1 |  |
| C1QTNF6 |  | LRP1 | VCAN |  | CACNA1C |  | VCAN |  | ZEB2 |  |
| CACNA1C |  | LRRC8C | WIPF1 |  | CCDC80 |  | WIPF1 |  |  |  |
| CCDC80 |  | MAF | ZEB2 |  | CCL11 |  | ZEB2 |  |  |  |

**Supplementary table 7: Comparison of performance by subtype of five gene lists in the GB validation dataset**

| **SRF50 (Overall Prediction Accuracy=80.1%)** | **Classical(N=50)** | | **Mesenchymal(N=48)** | **Neural(N=30)** | **Proneural(N=48)** |
| --- | --- | --- | --- | --- | --- |
| **Classical (N=50)** | | ***35 (70%)*** | 5 (10%) | 8 (16%) | 2 (4%) |
| **Mesenchymal (N=48)** | | 4 (8%) | ***39 (81%)*** | 2 (4%) | 3 (6%) |
| **Neural (N=30)** | | 2 (7%) | 1 (3%) | ***22 (73%)*** | 5 (17%) |
| **Proneural (N=48)** | | 2 (4%) | 0 (0%) | 1 (2%) | ***45 (94%)*** |
| **Single RF (Overall Prediction Accuracy=77.8%)** | | **Classical(N=50)** | **Mesenchymal(N=48)** | **Neural(N=30)** | **Proneural(N=48)** |
| **Classical (N=50)** | | ***32(64%)*** | 6(12%) | 11(22%) | 1(2%) |
| **Mesenchymal (N=48)** | | 5(10%) | ***37(77%)*** | 3(6%) | 3(6%) |
| **Neural (N=30)** | | 2(7%) | 1(3%) | ***24(80%)*** | 3(10%) |
| **Proneural (N=48)** | | 1(2%) | 0(0%) | 3(6%) | ***44(92%)*** |
| **Verhaak et al (Overall Prediction Accuracy=86.0%)** | | **Classical(N=50)** | **Mesenchymal(N=48)** | **Neural(N=30)** | **Proneural(N=48)** |
| **Classical (N=50)** | | ***44 (88%)*** | 5 (10%) | 0 (0%) | 1 (2%) |
| **Mesenchymal (N=48)** | | 2 (4%) | ***44 (92%)*** | 0 (0%) | 2 (4%) |
| **Neural (N=30)** | | 1 (3%) | 8 (27%) | ***15 (50%)*** | 6 (20%) |
| **Proneural (N=48)** | | 0 (0%) | 0 (0%) | 0 (0%) | ***48 (100%)*** |
| **ANOVA (Overall Prediction Accuracy=84.1%)** | | **Classical(N=50)** | **Mesenchymal(N=48)** | **Neural(N=30)** | **Proneural(N=48)** |
| **Classical (N=50)** | | ***44 (88%)*** | 4 (8%) | 0 (0%) | 2 (4%) |
| **Mesenchymal (N=48)** | | 2 (4%) | ***44 (92%)*** | 0 (0%) | 2 (4%) |
| **Neural (N=30)** | | 2 (7%) | 8 (27%) | ***12 (40%)*** | 8 (27%) |
| **Proneural (N=48)** | | 0 (0%) | 0 (0%) | 0 (0%) | ***48 (100%)*** |
| **Top 50 ANOVA (Overall Prediction Accuracy=77.2%)** | | **Classical(N=50)** | **Mesenchymal(N=48)** | **Neural(N=30)** | **Proneural(N=48)** |
| **Classical (N=50)** | | **32 (64%)** | 6 (12%) | 11 (22%) | 1 (2%) |
| **Mesenchymal (N=48)** | | 3 (6.25%) | **38 (79.17%)** | 4 (8.33%) | 3 (6.25%) |
| **Neural (N=30)** | | 3 (10%) | 2 (6.67%) | **22 (73.33%)** | 3 (10%) |
| **Proneural (N=48)** | | 1 (2.08%) | 0 (0%) | 3 (6.25%) | **44 (91.67%)** |

**Supplementary table 8: Pairwise AUC comparison between subtypes of five gene lists in the GB, BC and OC validation dataset**

| **Cancer** | **Pairwise AUC Comparison** | **SRF50** | **Single RF** | **Verhaak et al** | **ANOVA** | **Top 50 ANOVA** |
| --- | --- | --- | --- | --- | --- | --- |
| GB | Classical (N=50) VS Mesenchymal (N=48) | 0.74 | 0.71 | **0.91** | 0.9 | 0.73 |
|  | **Classical (N=50) VS Neural (N=30)** | 0.86 | 0.82 | **0.95** | **0.92** | 0.88 |
|  | Classical (N=50) VS Proneural (N=48) | 0.95 | 0.97 | 0.99 | 0.98 | **0.98** |
|  | **Mesenchymal (N=48) VS Neural (N=30)** | **0.85** | 0.79 | 0.81 | 0.78 | 0.85 |
|  | Mesenchymal (N=48) VS Proneural (N=48) | 0.93 | 0.94 | **0.98** | **0.98** | 0.94 |
|  | **Neural (N=30) VS Proneural (N=48)** | 0.87 | **0.91** | 0.9 | 0.87 | 0.87 |
|  | Multi-Class AUC | 0.87 | 0.86 | 0.92 | 0.9 | 0.87 |
| BC | **Basal-Like (N=18) VS HER2 (N=11)** | 0.91 | 0.91 | 0.95 | 0.91 | **1** |
|  | Basal-Like (N=18) VS Luminal A (N=20) | 1 | 1 | 1 | 1 | 1 |
|  | Basal-Like (N=18) VS Luminal B (N=32) | 1 | 1 | 1 | 1 | 1 |
|  | **HER2 (N=11) VS Luminal A (N=20)** | **0.84** | 0.68 | 0.65 | 0.58 | 0.68 |
|  | **HER2 (N=11) VS Luminal B (N=32)** | **0.88** | 0.79 | 0.81 | 0.76 | 0.78 |
|  | Luminal A (N=20) VS Luminal B (N=32) | 0.81 | 0.82 | **0.92** | **0.89** | 0.79 |
|  | Multi-Class AUC | 0.91 | 0.87 | 0.89 | 0.86 | 0.87 |
| OC | C1 (N=42) VS C2 (N=25) | 0.94 | 0.95 | 0.95 | **0.96** | 0.92 |
|  | C1 (N=42) VS C4 (N=23) | 0.98 | 1 | 1 | **1** | **0.99** |
|  | **C1 (N=42) VS C5 (N=18)** | 0.99 | **1** | **1** | **1** | 1 |
|  | C2 (N=25) VS C4 (N=23) | 0.88 | 0.9 | **0.92** | 0.88 | 0.9 |
|  | **C2 (N=25) VS C5 (N=18)** | **1** | 1 | 1 | 1 | 0.98 |
|  | **C4 (N=23) VSC5 (N=18)** | **0.96** | 0.91 | **0.95** | 0.96 | 0.89 |
|  | Multi-Class AUC | 0.96 | 0.96 | 0.97 | 0.97 | 0.95 |

*****Multi-Class AUC is derived by taking the average of all pairwise AUC values with GB, BC and OC for each method, respectively.

**Supplementary table 9: Hub genes in the top networks from IPA analysis for the SRF50 and published gene lists for GB, breast cancer (BC) and ovarian cancer (OC).**

| **GB** | | **BC** | | **OC** | |
| --- | --- | --- | --- | --- | --- |
| **SRF50_GB (N=19)** | **Verhaak et al (N=29)** | **SRF50_BC (N=19)** | **Parker et al (N=31)** | **SRF50_OC (N=31)** | **Tothill et al (N=32)** |
| CEBPA | LDL | ESR1 | NFkB | TGFBR2 | Gpcr |
| CKR | ABL1 | APC | BCL2 | ACTA1 | ACTA2 |
| ERK1/2 | BDNF | DLG4 | BIRC5 | Akt | ADCYAP1 |
| HNF4A | CASP1 | ERK1/2 | CCNB1 | APC | ADIPOQ |
| KITLG | CASP4 | GATA3 | CCNE1 | CDKN2A | Aip |
| MIR124 | CASP8 | HNF1A | CDC20 | COL1A1 | Akt |
| PDGFBB | CDK4/6 | HNF4A | CDC6 | COL1A2 | BMP4 |
| PI3K | CDKN2A | HTT | CDKN2A | COL3A1 | CD44 |
| PIK3R1 | CDKN2B | IL12 | CTNNB1 | ERK | CXCL10 |
| PLAUR | E2f | IL13 | EGFR | ERK1/2 | ERK1/2 |
| PPARG | ENO1 | IL6 | ERBB2 | FN1 | Fgfr |
| PTEN | ERBB | MAPK | ERBB3 | FSTL1 | FGFR1 |
| Rac | ERBB2 | MYC | ERK | FYN | FN1 |
| RB1 | ERK1/2 | NFkB | ERK1/2 | IL12 | FOXA2 |
| TGFB | HTT | NR3C1 | ESR1 | IL13 | ID1 |
| TNF | MET | P38MAPK | FZR1 | ITGA4 | IL1 |
| VEGF | MYC | PIK3 | KRAS | MAP2K1/2 | INHBA |
| WNT3A | PDGFBB | PIM1 | LDL | MMP11 | ITGB3 |
| ZBTB16 | PDGFRA | TREM1 | MAPT | MMP2 | LDL |
|  | PIK3CA |  | MDM2 | MYOD1 | MYOCD |
|  | PIK3R1 |  | MYBL2 | NFkB | NFkB |
|  | PTEN |  | MYC | PDGFBB | NRG1 |
|  | RB1 |  | PDGFBB | PDGFRB | P38MAPK |
|  | TGFB1 |  | PGR | PI3K | PDGFBB |
|  | TLR2 |  | PPARG | SPHK1 | PENK |
|  | TLR4 |  | PTTG1 | TGFB1 | PGR |
|  | TNFSRF1A |  | RB1 | THBS1 | PLAU |
|  | TOP1 |  | RHOA | TLR2/TLR4 | PLC gamma |
|  | TP53 |  | TGFB1 | TLR7 | RUNX2 |
|  |  |  | TP53 | TNFSF13B | SERPINE1 |
|  |  |  | UBE2C |  | SPARC |
|  |  |  |  |  | Vegf |

**Supplementary table 10: Comparison of performance by subtype of 3 gene lists in the Breast Cancer validation dataset**

|  | **Basal-Like (N=18)** | **HER2 (N=11)** | **Luminal A (N=20)** | **Luminal B (N=32)** |
| --- | --- | --- | --- | --- |
| **SRF50 (Overall Prediction Accuracy=84.0%)** |
| **Basal-Like (N=18)** | ***18(100%)*** | 0(0%) | 0(0%) | 0(0%) |
| **HER2 (N=11)** | 2(18%) | ***7(64%)*** | 0(0%) | 2(18%) |
| **Luminal A (N=20)** | 0(0%) | 0(0%) | ***16(80%)*** | 4(20%) |
| **Luminal B (N=32)** | 0(0%) | 1(3%) | 4(13%) | ***27(84%)*** |
| **Single RF (Overall Prediction Accuracy=77.8%)** | **Basal-Like (N=18)** | **HER2 (N=11)** | **Luminal A (N=20)** | **Luminal B (N=32)** |
| **Basal-Like (N=18)** | ***16 (89%)*** | 0 (0%) | 0 (0%) | 2 (11%) |
| **HER2 (N=11)** | 0 (0%) | ***8 (73%)*** | 3 (27%) | 0 (0%) |
| **Luminal A (N=20)** | 1 (5%) | 8 (40%) | ***11 (55%)*** | 0 (0%) |
| **Luminal B (N=32)** | 0 (0%) | 0 (0%) | 4 (13%) | ***28 (88%)*** |
| **Parker et al (Overall Prediction Accuracy=89.0%)** | **Basal-Like (N=18)** | **HER2 (N=11)** | **Luminal A (N=20)** | **Luminal B (N=32)** |
| **Basal-Like (N=18)** | ***18 (100%)*** | 0 (0%) | 0 (0%) | 0 (0%) |
| **HER2 (N=11)** | 1 (9%) | ***6 (55%)*** | 0 (0%) | 4 (36%) |
| **Luminal A (N=20)** | 0 (0%) | 0 (0%) | ***18 (90%)*** | 2 (10%) |
| **Luminal B (N=32)** | 0 (0%) | 0 (0%) | 2 (6%) | ***30 (94%)*** |
| **ANOVA (Overall Prediction Accuracy=85.2%)** | **Basal-Like (N=18)** | **HER2 (N=11)** | **Luminal A (N=20)** | **Luminal B (N=32)** |
| **Basal-Like (N=18)** | ***18 (100%)*** | 0 (0%) | 0 (0%) | 0 (0%) |
| **HER2 (N=11)** | 2 (18%) | ***4 (36%)*** | 0 (0%) | 5 (45%) |
| **Luminal A (N=20)** | 0 (0%) | 0 (0%) | ***17 (85%)*** | 3 (15%) |
| **Luminal B (N=32)** | 0 (0%) | 0 (0%) | 1 (3%) | ***30 (94%)*** |
| **Top 50 ANOVA (Overall Prediction Accuracy=82.7%)** | **Basal-Like (N=18)** | **HER2 (N=11)** | **Luminal A (N=20)** | **Luminal B (N=32)** |
| **Basal-Like (N=18)** | ***18 (100%)*** | 0 (0%) | 0 (0%) | 0 (0%) |
| **HER2 (N=11)** | 0 (0%) | ***7 (64%)*** | 0 (0%) | 4 (36%) |
| **Luminal A (N=20)** | 0 (0%) | 0 (0%) | ***15 (75%)*** | 5 (25%) |
| **Luminal B (N=32)** | 0 (0%) | 1 (3%) | 4 (13%) | ***27 (84%)*** |

**Supplementary table 11: Comparison of performance by subtype of 3 gene lists in the Ovarian Cancer validation dataset**

| **SRF50 (Overall Prediction Accuracy=89.8%)** | **C1 (N=42)** | **C2 (N=25)** | **C4 (N=23)** | **C5 (N=18)** |
| --- | --- | --- | --- | --- |
| **C1 (N=42)** | ***41 (98%)*** | 0 (0%) | 0 (0%) | 1 (2%) |
| **C2 (N=25)** | 2 (8%) | ***22 (88%)*** | 1 (4%) | 0 (0%) |
| **C4 (N=23)** | 0 (0%) | 5 (22%) | ***17 (74%)*** | 1 (4%) |
| **C5 (N=18)** | 0 (0%) | 0 (0%) | 1 (6%) | ***17 (94%)*** |
| **Single RF (Overall Prediction Accuracy=88.9%)** | **C1 (N=42)** | **C2 (N=25)** | **C4 (N=23)** | **C5 (N=18)** |
| **C1 (N=42)** | ***41 (98%)*** | 1 (2%) | 0 (0%) | 0 (0%) |
| **C2 (N=25)** | 2 (8%) | ***22 (88%)*** | 1 (4%) | 0 (0%) |
| **C4 (N=23)** | 0 (0%) | 4 (17%) | ***18 (78%)*** | 1 (4%) |
| **C5 (N=18)** | 0 (0%) | 0 (0%) | 3 (17%) | ***15 (83%)*** |
| **Tothill et al (Overall Prediction Accuracy=91.7%)** | **C1 (N=42)** | **C2 (N=25)** | **C4 (N=23)** | **C5 (N=18)** |
| **C1 (N=42)** | ***41 (98%)*** | 1 (2%) | 0 (0%) | 0 (0%) |
| **C2 (N=25)** | 2 (8%) | ***22 (88%)*** | 1 (4%) | 0 (0%) |
| **C4 (N=23)** | 0 (0%) | 3 (13%) | ***20 (87%)*** | 0 (0%) |
| **C5 (N=18)** | 0 (0%) | 0 (0%) | 2 (11%) | ***16 (89%)*** |
| **ANOVA (Overall Prediction Accuracy=90.7%)** | **C1 (N=42)** | **C2 (N=25)** | **C4 (N=23)** | **C5 (N=18)** |
| **C1 (N=42)** | ***42 (100%)*** | 0 (0%) | 0 (0%) | 0 (0%) |
| **C2 (N=25)** | 2 (8%) | ***22 (88%)*** | 1 (4%) | 0 (0%) |
| **C4 (N=23)** | 0 (0%) | 5 (22%) | ***18 (78%)*** | 0 (0%) |
| **C5 (N=18)** | 0 (0%) | 0 (0%) | 2 (11%) | ***16 (89%)*** |
| **Top 50 ANOVA ( (Overall Prediction Accuracy=87.0%)** | **C1 (N=21)** | **C2 (N=13)** | **C4 (N=12)** | **C5 (N=18)** |
| **C1 (N=21)** | ***40 (95%)*** | 2 (5%) | 0 (0%) | 0 (0%) |
| **C2 (N=13)** | 3 (12%) | ***22 (88%)*** | 0 (0%) | 0 (0%) |
| **C4 (N=12)** | 0 (0%) | 5 (22%) | ***17 (74%)*** | 1 (4%) |
| **C5 (N=18)** | 0 (0%) | 1 (6%) | 2 (11%) | ***15 (83%)*** |

**Supplementary Figure Legend**

Supplementary Figure 1 A, B, C: Venn diagram of the gene lists from SRF50, 100 and 500 runs for GB, breast cancer and ovarian cancer

Supplementary Figure 2: Histograms of the SRF50, 100 and 500 runs predictions by subtype for GB

Supplementary Figure 3: Histograms of the SRF50, 100 and 500 runs predictions by subtype for breast cancer

Supplementary Figure 4: Histograms of the SRF50, 100 and 500 runs predictions by subtype for ovarian cancer

Supplementary Figure 5: Histograms of the SRF, Verhaak and ANOVA validation by subtype for GB

Supplementary Figure 6 A and B: IPA network plots showing the overlap in the hub genes for the SRF50 gene list and the Verhaak list (grey lines denote the SRF50 gene list and green lines denote the Verhaak gene list)

Supplementary Figure 7: Histograms of the SRF, Parker and ANOVA validation by subtype for breast cancer

Supplementary Figure 8 A, B and C: IPA network plots showing the overlap in the hub genes for the SRF50 gene list and the Parker list (grey lines denote the SRF50 gene list and green lines denote the Parker gene list)

Supplementary Figure 9: Histograms of the SRF, Tothill and ANOVA validation by subtype for ovarian cancer

Supplementary Figure 10 A, B, C and D: IPA network plots showing the overlap in the hub genes for the SRF50 gene list and the Tothill list (grey lines denote the SRF50 gene list and green lines denote the Tothill gene list)

**Supplementary Figure 1**

1. **B.**

**
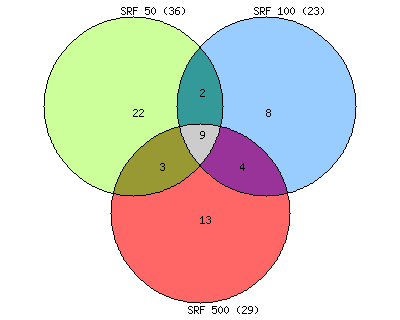

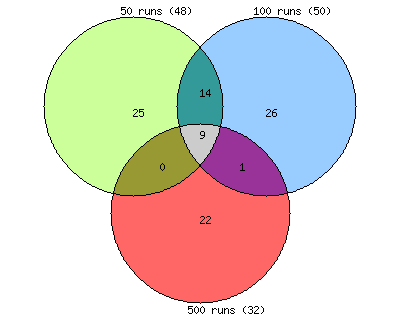
**

**C.**

**
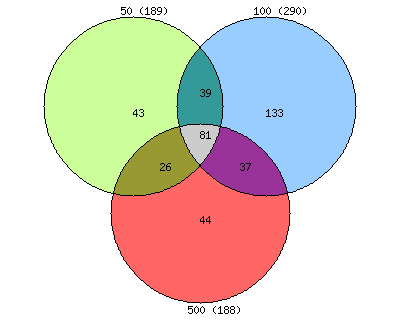
**

**Supplementary Figure 2**

**
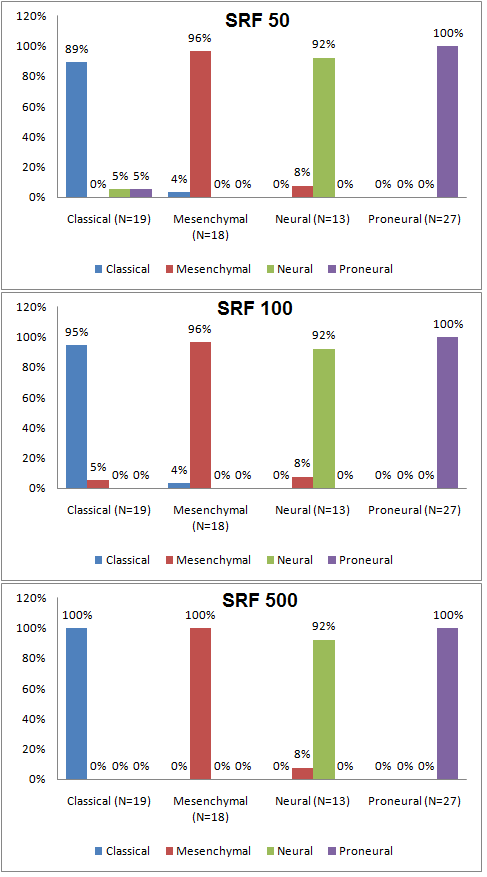
**

**Supplementary Figure 3**

**
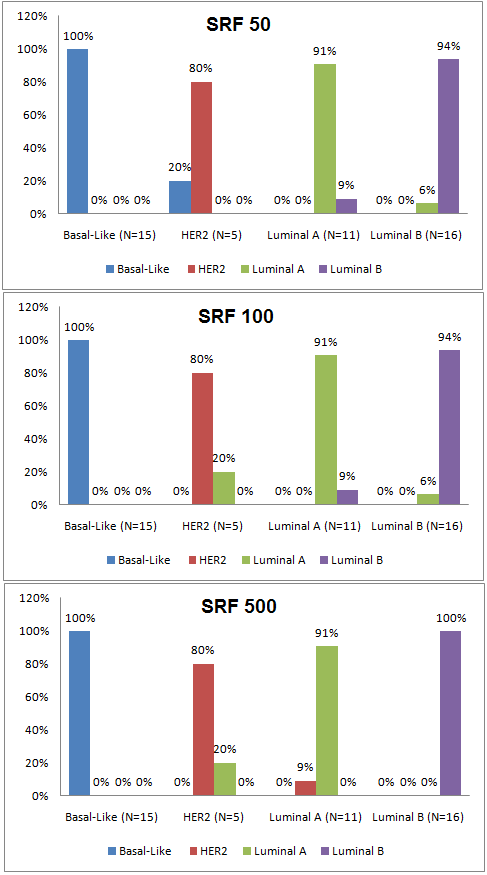
**

**Supplementary Figure 4**

**
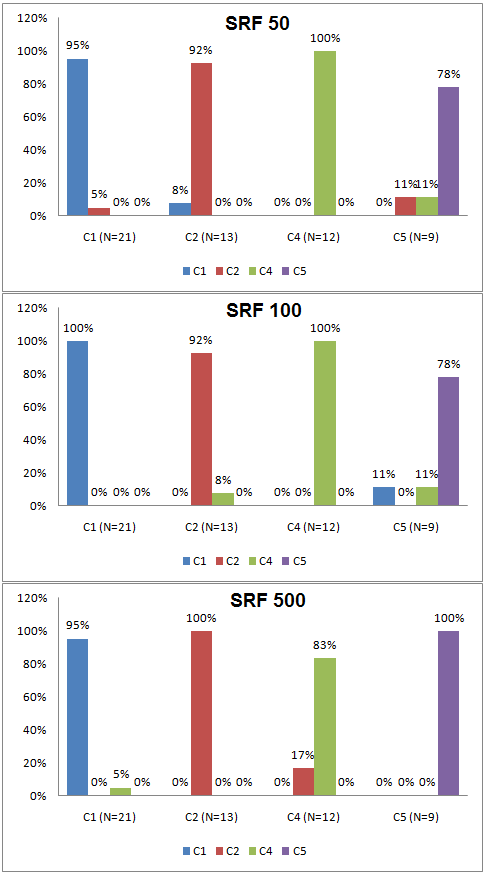
**

**Supplementary Figure 5**

**
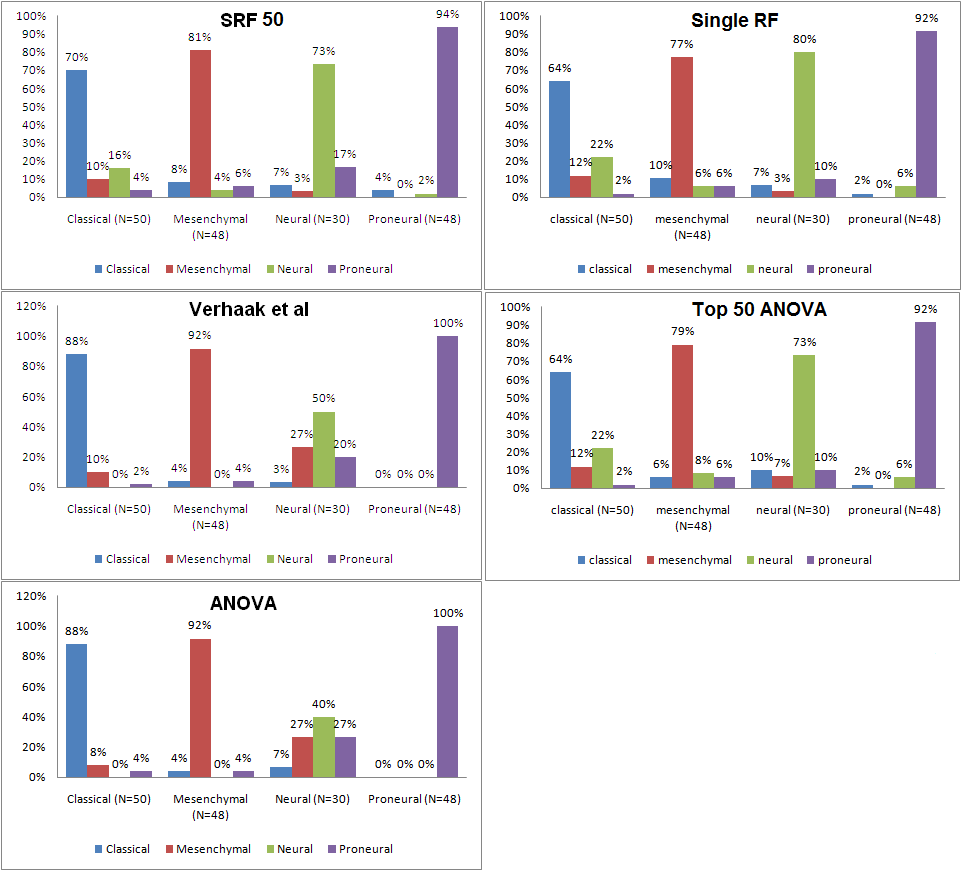
**

**Supplementary Figure 6A**

**
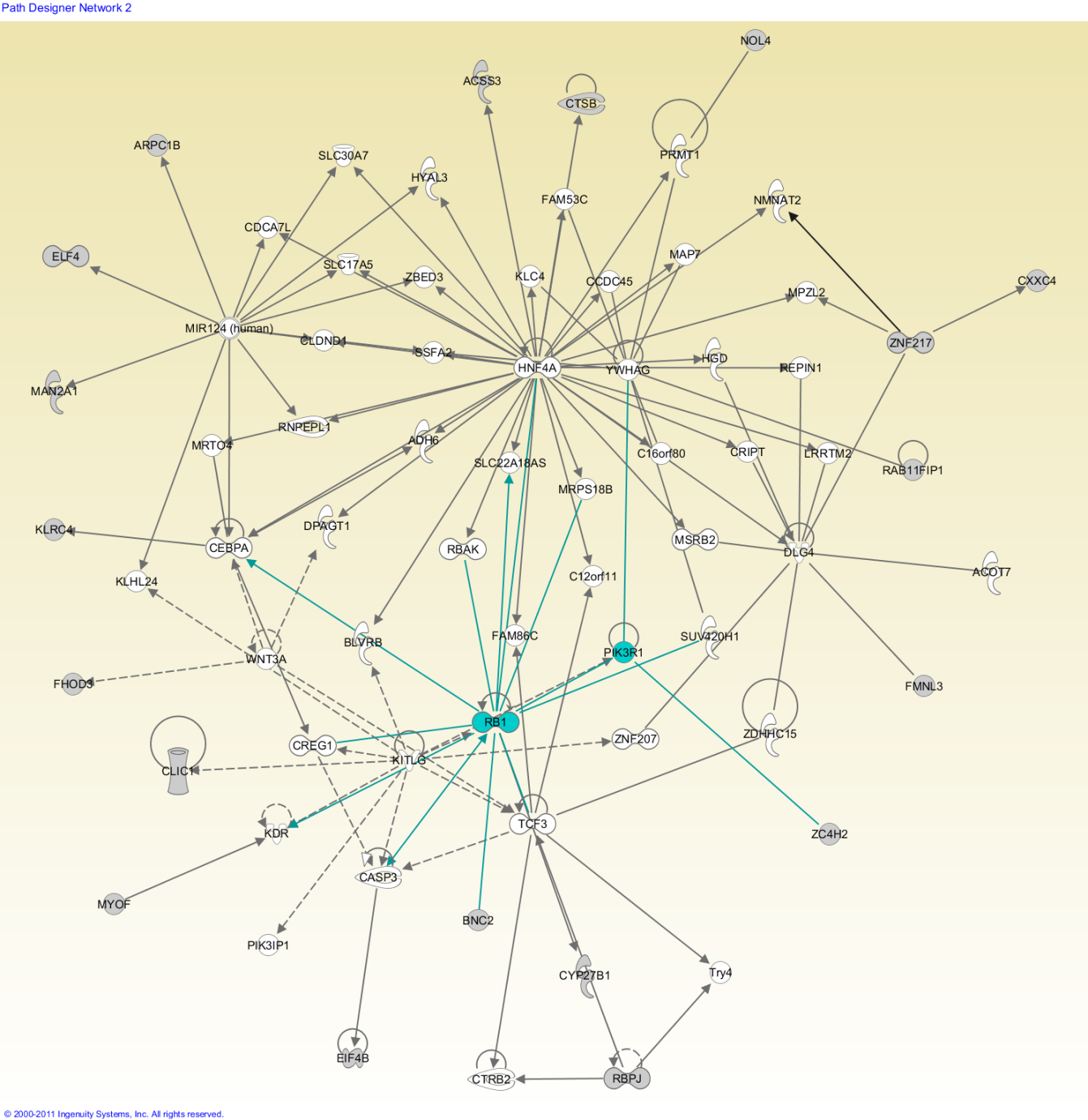
**

**Supplementary Figure 6B**

**
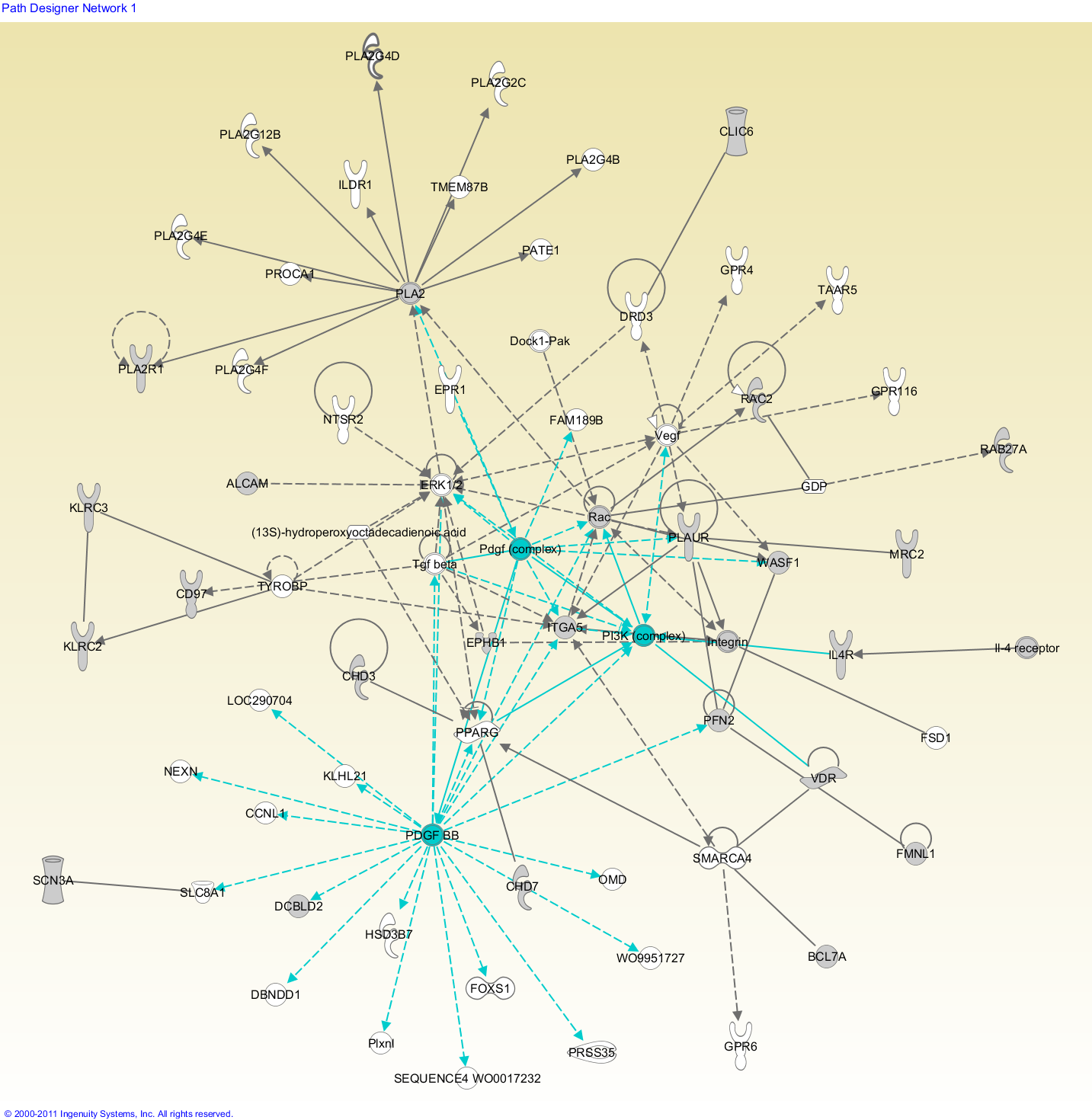
**

**Supplementary Figure 7**

**
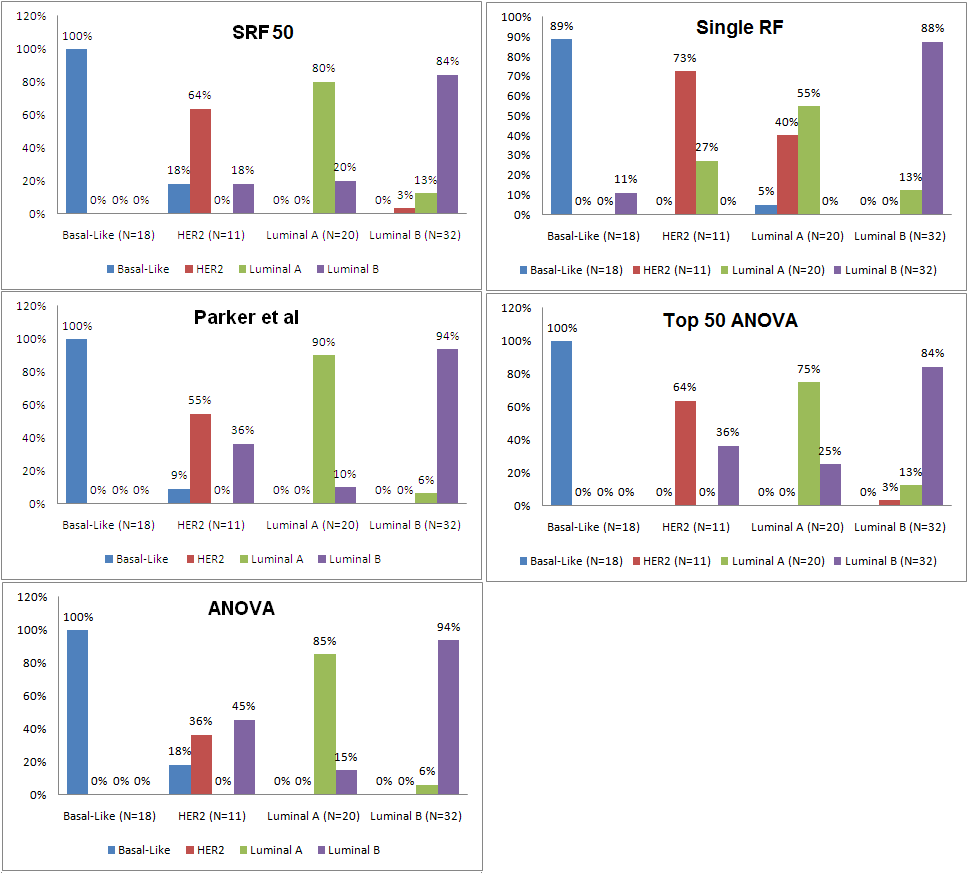
**

**Supplementary Figure 8A**


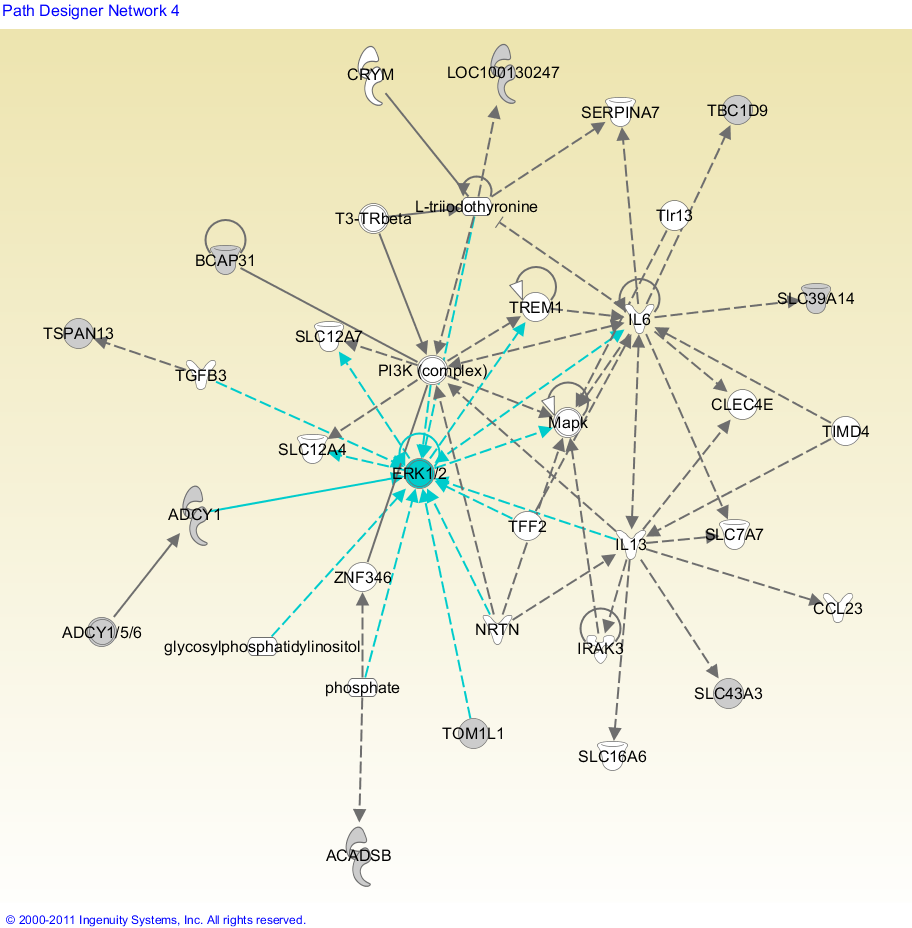


**Supplementary Figure 8B**


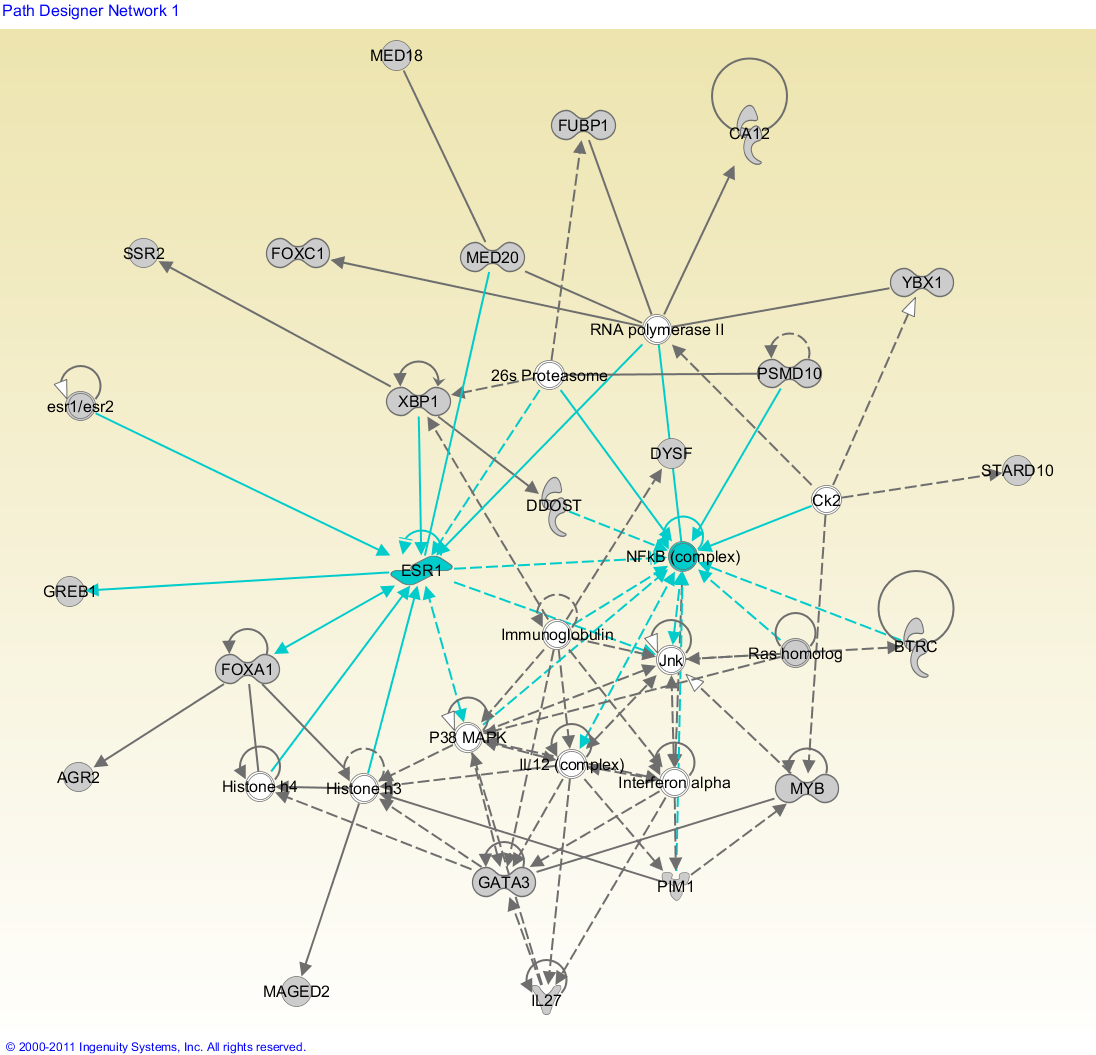


**Supplementary Figure 8C**


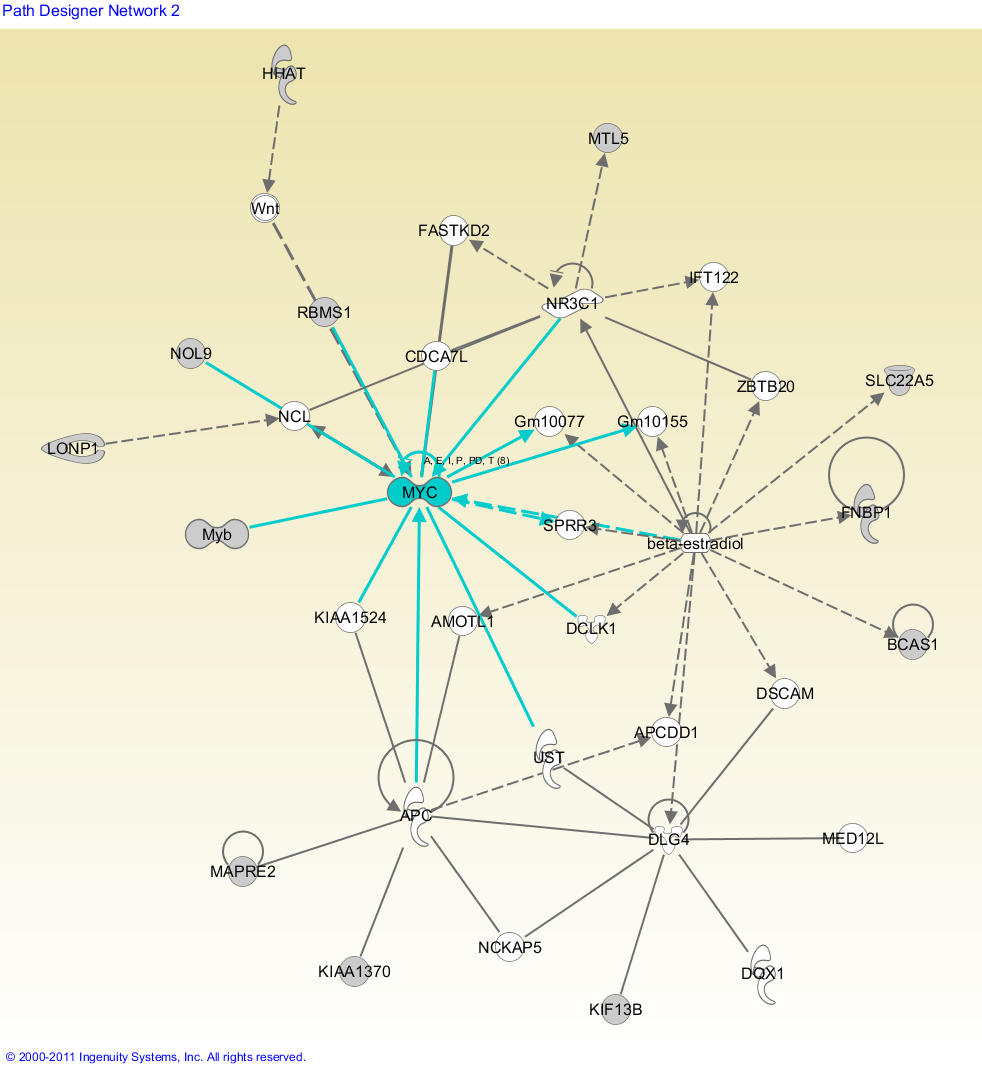


**Supplementary Figure 9**

**
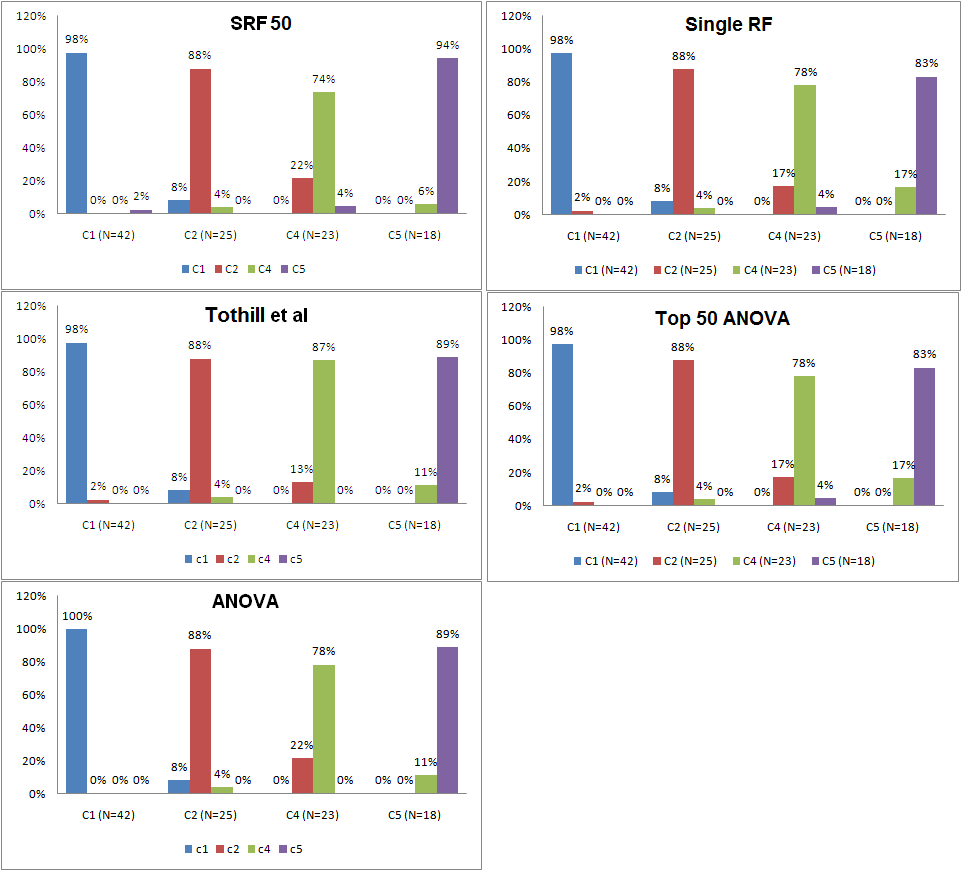
**

**Supplementary Figure 10A**

**
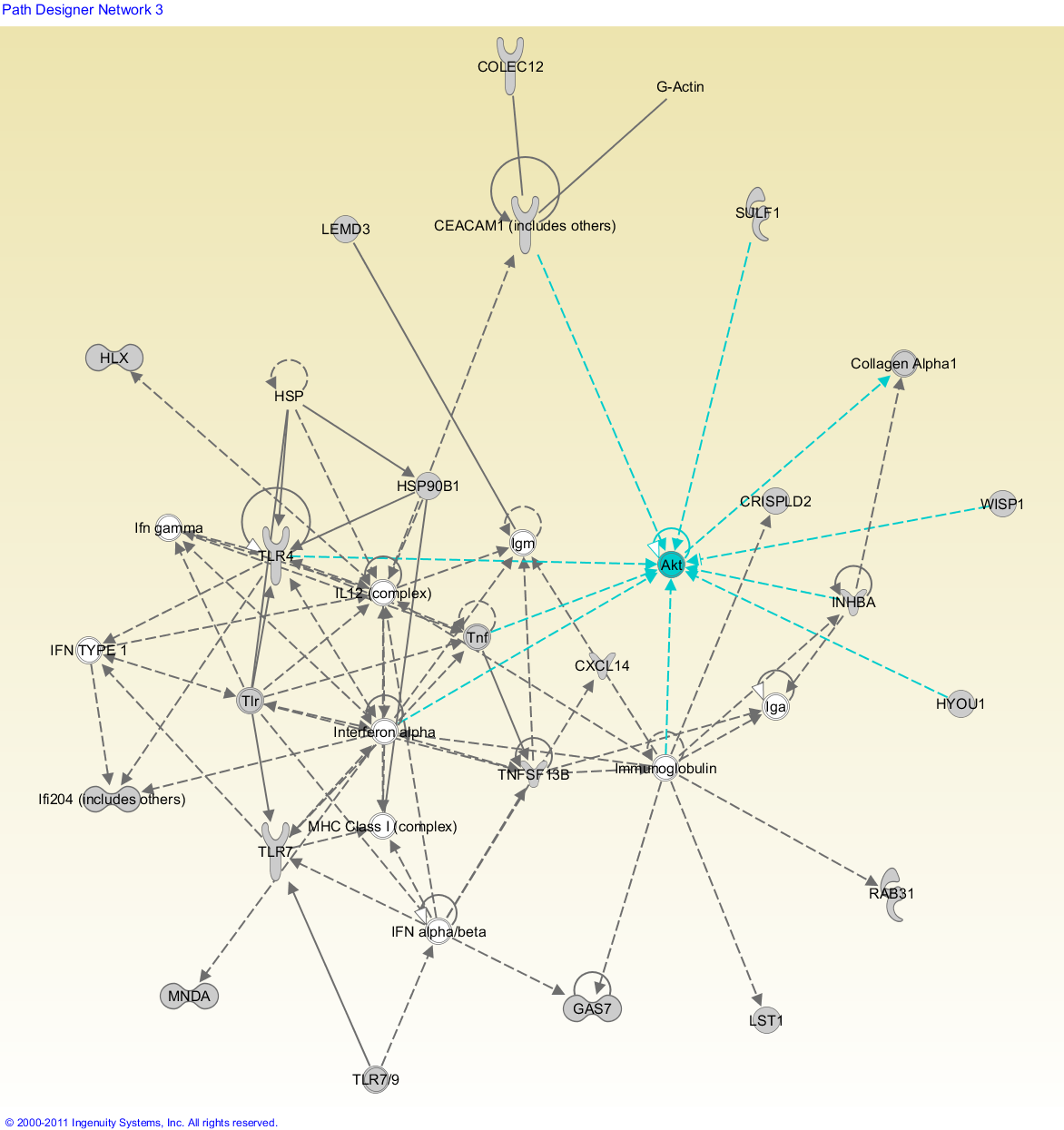
**

**Supplementary Figure 10B**

**
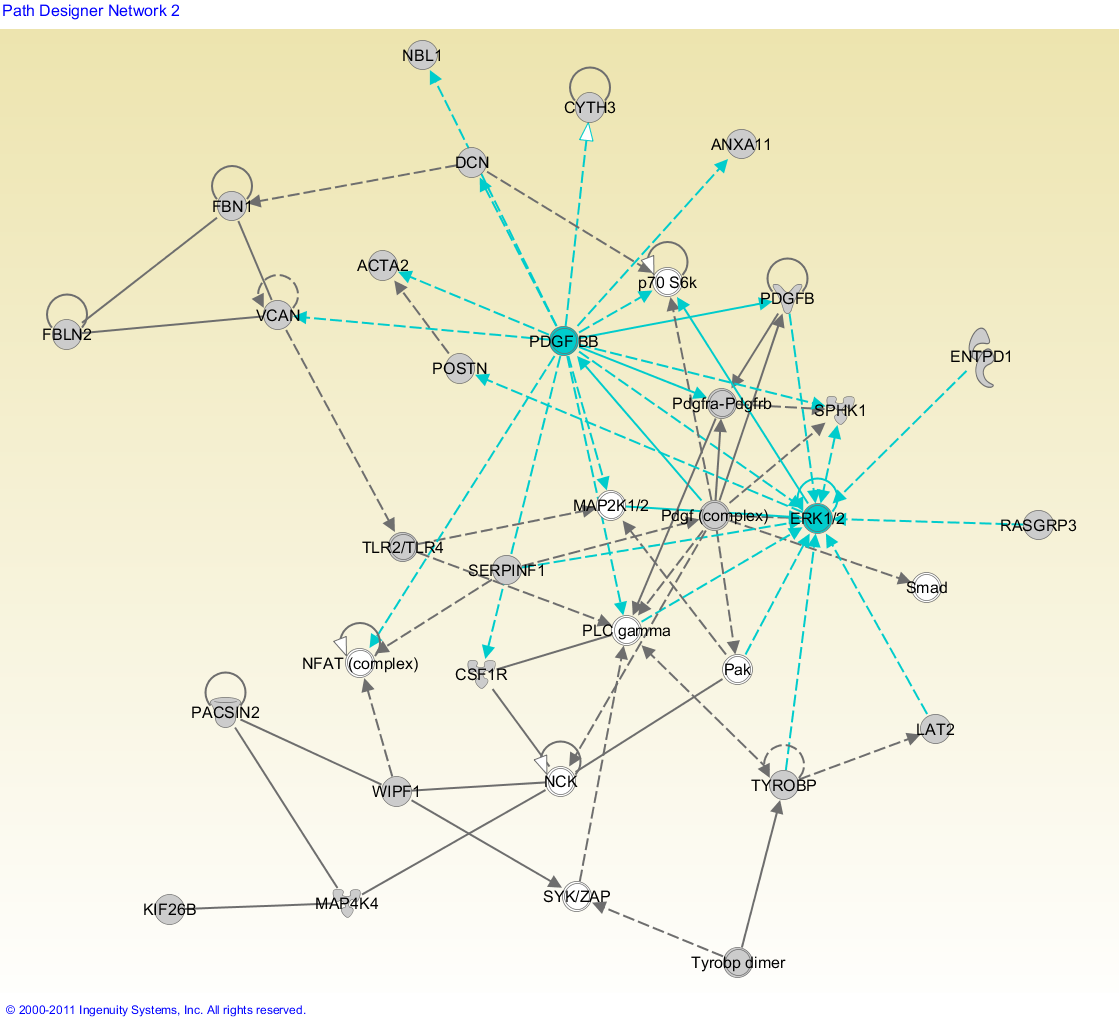
**

**Supplementary Figure 10C**

**
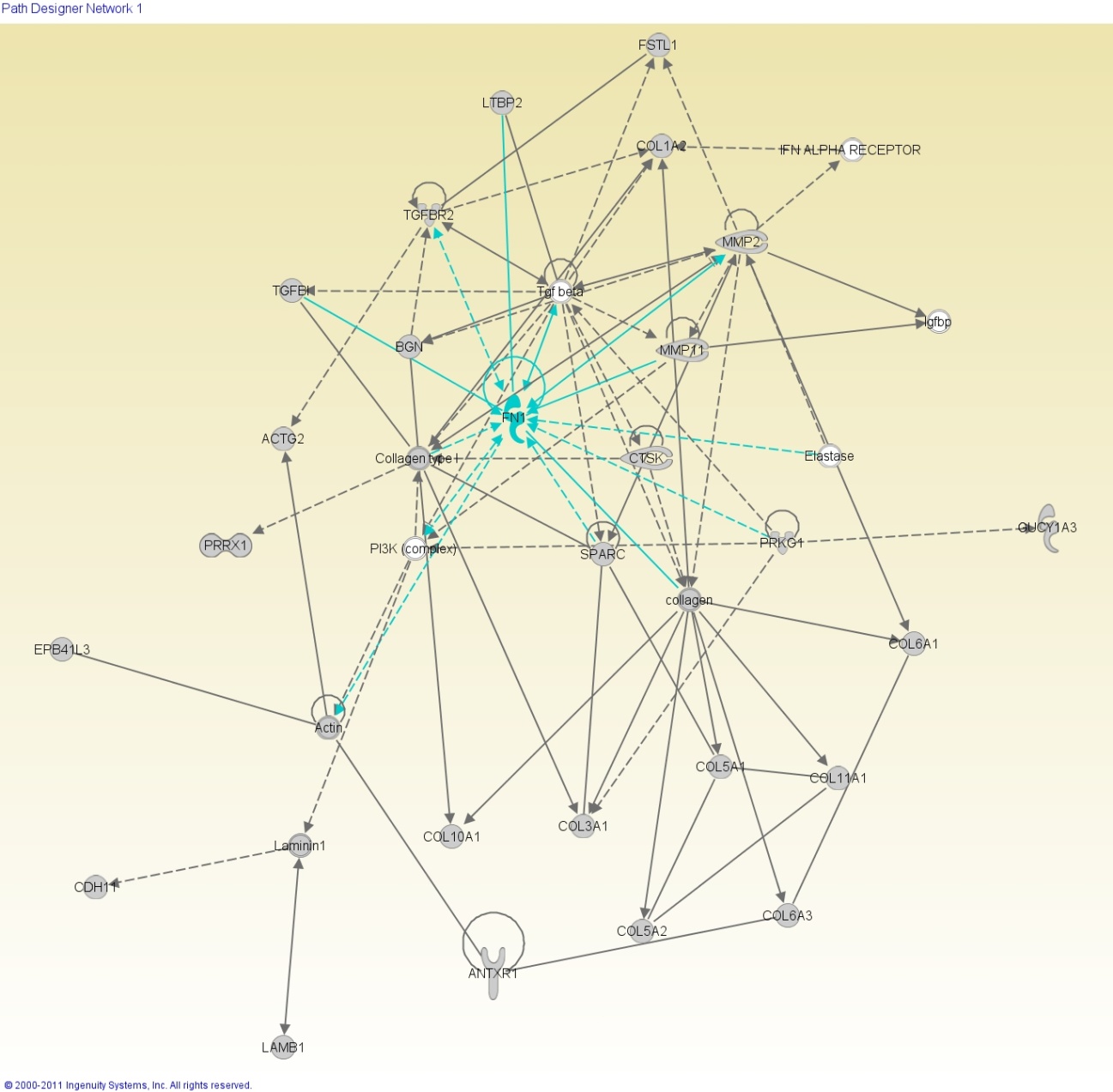
**

**Supplementary Figure 10D**

**
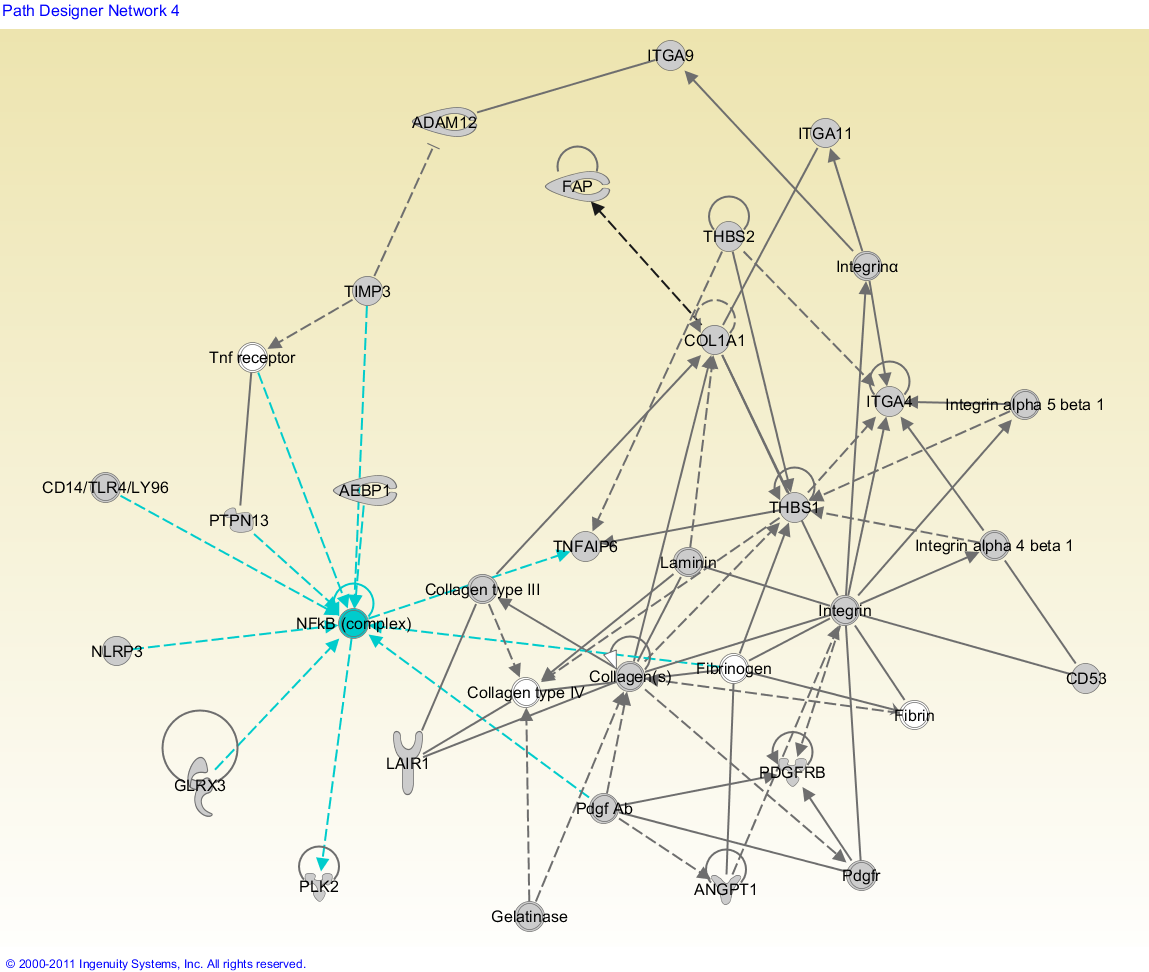
**
